# A minimized symbiotic gene set from the 1.68 Mb pSymB chromid of *Sinorhizobium meliloti* reveals auxiliary symbiotic loci

**DOI:** 10.1186/s12915-025-02298-5

**Published:** 2025-07-09

**Authors:** Jason V. S. Kearsley, Barney A. Geddes, George C. diCenzo, Maryam Zamani, Turlough M. Finan

**Affiliations:** 1 Present Address: Department of Biology, McMaster University, 1280 Main St. W., Hamilton, Ontario, L8S 4K1 Canada; 2 Present Address: Department of Microbiological Sciences, North Dakota State University, 1523 Centennial Blvd, ND Fargo, 58102 USA; 3 Present Address: Department of Biology, Queens University, 116 Barrie St., Kingston, Ontario, K7L 3N6 Canada

**Keywords:** Minimal genome, Symbiotic nitrogen-fixation (SNF); Root nodule symbiosis, Chromid, Megaplasmid, Legume-rhizobia symbiosis

## Abstract

**Background:**

Symbiotic nitrogen-fixation between bacteria called rhizobia and leguminous plants is a critical aspect of sustainable agriculture. Complex, two-way communication governs the invasion of plant roots and the formation of nodules in which the rhizobia reduce N_2_ to bioavailable ammonia. Research has uncovered many of the genes required for the symbiosis; however, engineering the symbiosis to function with alternative hosts such as cereal crops necessitates the establishment of a core set of symbiotic players.

**Results:**

We examined the symbiotic relevance of the genes on the 1.68 Mb pSymB chromid of the model rhizobium *Sinorhizobium meliloti*. By employing a strain in which pSymB was removed, we used a gain-of-function approach to assess a select group of known symbiotic regions totalling 261 kb (15.5%) of pSymB. This gene set enabled symbiotic N_2_-fixation with alfalfa with a high degree of plant genotype-dependent variation in which nodules often senesced prematurely. We demonstrate that additional regions lacking canonical symbiosis genes are important for the efficient formation of symbiosis with the plant host. These regions appear to contain auxiliary symbiotic loci whose genes encode products with quasi-essential functions for the symbiosis and that are redundant in nature. We further established a 673-kb pSymB genome that engages consistently in N_2_-fixation with alfalfa with 45% efficiency.

**Conclusions:**

The reduction of the pSymB genome showcases the complexity and nuance of its involvement in the N_2_-fixing symbiosis.

**Supplementary Information:**

The online version contains supplementary material available at 10.1186/s12915-025-02298-5.

## Background

Reactive nitrogen (Nr), or fixed nitrogen, reduced from its molecular form (N_2_) is an essential component of nucleic acids, proteins, and many other biomolecules. The Haber–Bosch process—the synthesis of ammonia from its elements—is arguably responsible for the explosion in global population following its 1908 discovery [1, 2]. However, the unsustainable application of Nr fertilizers has polluted the biosphere with an abundance of nitrogenous compounds [3, 4]. Prior to the advent of Haber–Bosch Nr, biological nitrogen fixation (BNF) represented 90% of terrestrially fixed N_2_ [5]. Anthropogenic Nr production continues to increase annually (210 Tg year^−1^), eclipsing that of natural sources (203 Tg year^−1^) and effectively doubling the global cycling of nitrogen [6, 7].

A wide range of diazotrophs (exclusively bacteria and archaea), carry out BNF via a strongly conserved O_2_-sensitive metalloenzyme, nitrogenase, which catalyzes the energy intensive reduction of N_2_ to NH_3_ [8, 9]. BNF accounts for 50–70 Tg of Nr in agricultural systems, with ~ 20 Tg stemming from legume crops [10]. Leguminous plants are noteworthy for forming a symbiotic interaction with two classes of *Pseudomonadota* (syn. *Proteobacteria*)—*Alphaproteobacteria* and *Betaproteobacteria*—which are collectively termed rhizobia. The interaction commences in the soil, where rhizobia are in competition for rhizosphere occupancy. A complex and not yet fully understood signal exchange between the bacteria and its prospective host plant guides the growth and division of the rhizobia down structures called infection threads [5, 11]. The bacteria are then released into the cortical cells of the nodule where they are surrounded by a plant membrane and differentiate into bacteroids, forming what is known as the symbiosome [12, 13]. The bacteroid is the site of nitrogenase-mediated N_2_ fixation and the resultant ammonium is subsequently assimilated by the host plant at the expense of some of its photosynthate and the resources spent for nodule development [14].

Decades of research have uncovered hundreds of genes involved in the symbiotic nitrogen fixation (SNF) process in both the bacteria and host plant [15, 16]. Interest in rhizobia that fix nitrogen has been spurred by the possibility of introducing the symbiosis into non-leguminous plants, primarily cereals such as rice and wheat [17]. One approach involves directly transferring the nitrogenase genes into plant cells [18, 19]. Another approach revolves around engineering the symbiosis to work within other hosts [20]. Both approaches require a deep understanding of the genetics at play to successfully achieve the desired phenotype [21].

The reliance of the symbiosis on a complex exchange of chemical signals and coordinated responses between both organisms demands the simplest possible solution [22, 23]. It is therefore important to establish the minimal nitrogen fixing genome for a model system of a specific bacterium and its host organism. The question of how many rhizobial genes are needed for nodule development and function has been asked for some time [24]. Minimal and synthetic genome projects have typically involved reducing genetic content while maintaining cell viability or streamlining the genome by recoding and removing superfluous factors like insertion elements [25–28]. Such approaches and design philosophies are easily extended to a process such as SNF. Many essential genes for the symbiosis have been identified and characterized; however, there are undeniably more genes whose removal has a significant but not catastrophic impact on the symbiosis—what Hutchison III et al. [26] have called quasi-essential genes [29]. The model rhizobial symbiont *Sinorhizobium meliloti* is well studied within its model host *Medicago truncatula* and the closely related crop plant *Medicago sativa* (alfalfa) [11]. The most characterized *S. meliloti* strains Rm1021 and Rm2011 possesses a 6.7 Mb tripartite genome consisting of a 3.65 Mb chromosome, the 1.35 Mb pSymA megaplasmid, and 1.68 Mb pSymB chromid [30]. Many of the genes required for SNF are located on the two extrachromosomal replicons [31].

The pSymA megaplasmid is conventionally regarded as the symbiotic megaplasmid because it harbours the *nod*, *nif*, and *fix* genes that encode the critical signalling molecule (Nod factor), nitrogenase, and proteins that confer metabolic adaptation to the microaerobic nodule environment (including electron transport to nitrogenase), respectively [32]. Its accessory nature can best be highlighted by the observation that it can be removed without compromising cell viability [33]. Unsurprisingly, disruption or removal of the SNF genes on pSymA results in phenotypes deficient in nitrogen fixation (Fix^−^) and/or nodule formation (Nod^−^) [34–36]. Our group used this information to design and construct an initial minimal pSymA gene set of 162 kb that was capable of robust SNF, which was further reduced to a mere 58 protein coding genes (62.7 kb) [37].

The pSymB chromid also harbours genes known to be required for SNF, albeit less overtly involved than those on pSymA [38]. The most studied are genes responsible for the synthesis of the symbiotically important exopolysaccharide (EPS) succinoglycan that is required for infection thread formation and elongation [39]. Evidence suggests pSymB is evolutionarily older than pSymA, and part of its genome appears to have translocated from the chromosome [40–42]. This translocated region, referred to as ETR (*engA*-*tRNA*^ARG^-*rmlC*), is bordered on one end by the essential *engA* gene encoding a GTPase involved in ribosome biogenesis and at the other end by *rmlC*. The *Sinorhizobium* ETR region is variable in size between species and contains a second essential gene—a tRNA^ARG^ that recognizes the CGG codon [40]. Perhaps the notion that pSymB is more integrated into the *S. meliloti* genome than pSymA is best highlighted by observations that its removal results in a slower rate of growth in minimal and complex media as well as bulk soil [43]. Such megaplasmids have been termed chromids given their more chromosomal-like properties [44]. Like the aforementioned pSymA deletions, there are several regions on pSymB whose deletion completely abolished SNF with *M. sativa* (Fix^−^). Of fifteen total large-scale deletions spanning the entirety of pSymB, three, ΔB108, ΔB109, and ΔB123, representing 196-kb and less than 12% of the total pSymB sequence, displayed severe deficits in symbiosis [29]. The initial goal of this work was to construct a strain in which these three regions were combined and re-introduced into a suitable background strain lacking pSymB.

Here we report findings from experiments designed to establish the minimum complement of pSymB genes that are sufficient for the formation of robust symbiotic nitrogen-fixing nodules with alfalfa. We initially constructed a strain in which the B108, B109, and B123 regions were combined and introduced into a suitable background strain lacking pSymB that was previously engineered to include the 69-kb ETR region from *Sinorhizobium fredii* [29]. This strain contained 261 kb of pSymB DNA, representing a reduction of 84.5% of the total pSymB sequence. We subsequently characterized its variable effectiveness at forming robust SNF with *M. sativa* and then further explored the involvement of additional pSymB genes in SNF through deletion analyses. Our work highlights the complexity of the symbiotic genome and exposes redundancies between loci of considerable interest.

## Results

To experimentally define a minimal pSymB gene set required for symbiosis, we used an *S. meliloti* strain that carried a chromosomal insertion of the 69-kb ETR region, including the essential *engA* and *tRNA*^ARG^, from *S. fredii* NGR234. Sixty-one of the sixty-seven genes contained within this region have probable orthologs in the larger 129-kb *S. meliloti* counterpart and constitute a portion of the minimal symbiotic gene set (Additional file 1: Table S1). The *S. fredii* (*Sf*) *bacA* gene within the ETR region was replaced with the *S. meliloti* (*Sm*) *bacA* as it was previously found to be required for an effective symbiosis with alfalfa [45]. Lastly, the complete pSymB replicon was removed and the resulting *S.meliloti* ΔpSymB strain was employed in the experiments below. This strain was designated RmP3952 and its genotype was Rm2011 ΩETR(*Sf*) Δ*bacA*(*Sf*)::*bacA*(*Sm*) ΔpSymB.

The host plant used in this study was *M. sativa* (alfalfa) as the wild-type *S. meliloti* strains employed here form highly effective root nodules and are known to form a poor symbiosis with the model legume *M. truncatula* Jemalong A17 [46]. As noted below, as an alternative plant host, we also tested the symbiotic phenotype of some strains with *Melilotus officinalis* (yellow sweet clover).

### Construction of minSymB1.0—an initial minimized pSymB symbiotic gene set

**Fig. 1:**
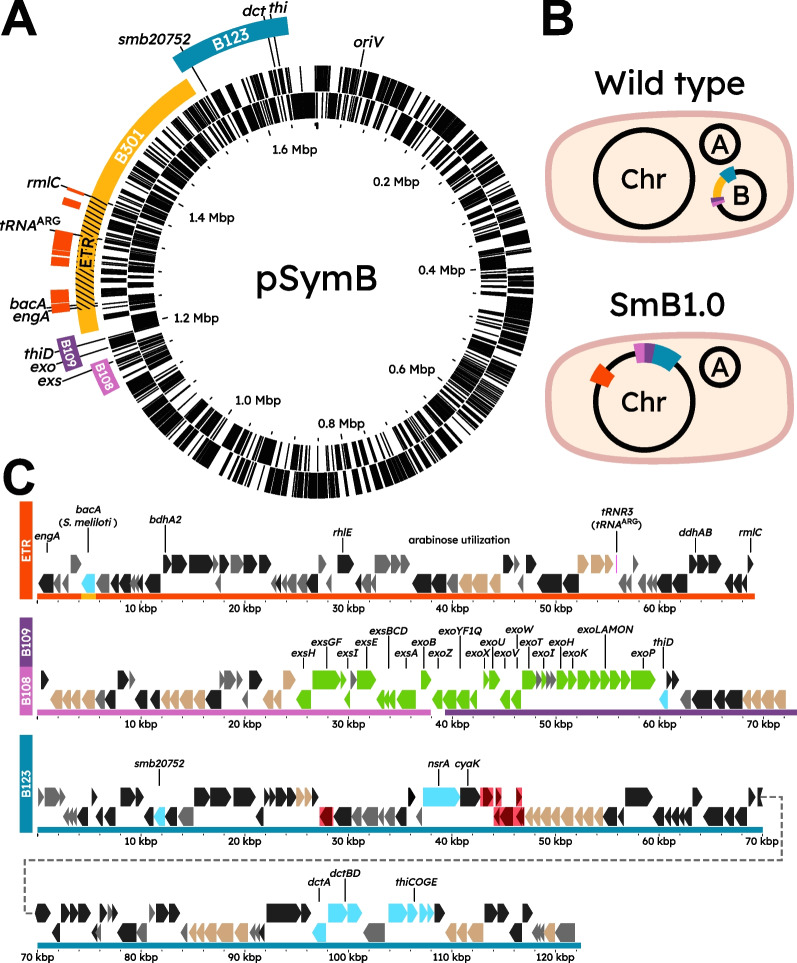
Map of *S. meliloti *pSymB and regions included in the initial minimized gene set, minSymB1.0.** A** Full wild-type pSymB with B108, B109, and B123 regions identified by diCenzo et al. (2016) as being absolutely critical for SNF with alfalfa. Relevant canonical and other important genes for efficient SNF are highlighted along with the essential *engA* and *tRNA*^ARG^ genes within the ETR (hashed) of the B301 region (yellow). *S. fredii* NGR234 homologues to *S. meliloti* ETR genes are depicted (orange). ***exo***: *exoA,B,F1,H,I,K-Q,T-Z*, ***exs***: *exsA-I*, ***dct***: *dctA,B,D*, ***thi***: *thiC,O,G,E*. **B** Schematic (not to scale) of the chromosome and megaplasmids present in wild-type *S. meliloti* Rm2011 versus the minSymB1.0 strain SmB1.0. Note that SmB1.0 lacks a replicating pSymB and contains the ETR region from *S. fredii* NGR234 on its chromosome (orange) along with the B108-B109-B123 regions from pSymB. Importantly the *bacA* gene of the *S. fredii* ETR (orange) has been replaced by the *S. meliloti* copy (yellow). **C** Detailed map of the regions and the genes (represented as arrows) contained within minSymB1.0 when integrated into the chromosome of a ΔpSymB strain. The genes involved in succinoglycan biosynthesis are indicated in green. Other relevant SNF related genes are coloured cyan. Genes encoding ABC transporters are shown as beige, while genes encoding proteins with predicted functions and hypothetical proteins are shown as black and grey, respectively. Genes highlighted in red indicate foreign insertion elements

A previous deletion analysis of pSymB identified three regions, B108, B109, and B123 that were required for the formation of N_2_-fixing (Fix^+^) nodules with alfalfa [29]. B108-B109 is a contiguous DNA fragment separated from the B123 region by the 326-kb B301 region (Fig. 1A). As an initial minimal assemblage of symbiotic genes, we wished to determine the symbiotic phenotype of an *S. meliloti* ΔpSymB strain carrying the B108, B109, and B123 regions. To construct this strain, the B301 region was deleted to bring the three regions together and the resulting B108-B109-B123 fragment was excised and captured in *E. coli* as the replicating plasmid pTH3247 (Additional file 2: Fig. S1). This plasmid was then integrated into a ΦC31-*attB* landing pad in the chromosome of the *S. meliloti* ΔpSymB strain (RmP3952). The resultant strain [ΩETR(*Sf*)Δ*bacA*(*Sf*)::*bacA*(*Sm*) ΩB108-B109-B123, ΔpSymB] was designated RmP4256 (hereafter termed SmB1.0), and its pSymB content is henceforth referred to as minSymB1.0. A schematic of the genomic content of this strain compared to a wild-type *S. meliloti* strain is summarized in Fig. 1B. Including the ETR region integrated into the chromosome (and assuming the *S. fredii* ETR gene copies can function in lieu of their *S. meliloti* orthologs), SmB1.0 possesses 261 kb of pSymB DNA, representing 15.5% of the chromid (Fig. 1C).

### The minSymB1.0 gene complement supports normal nodulation and is sufficient for SNF with alfalfa with markedly reduced efficiency

**Fig. 2:**
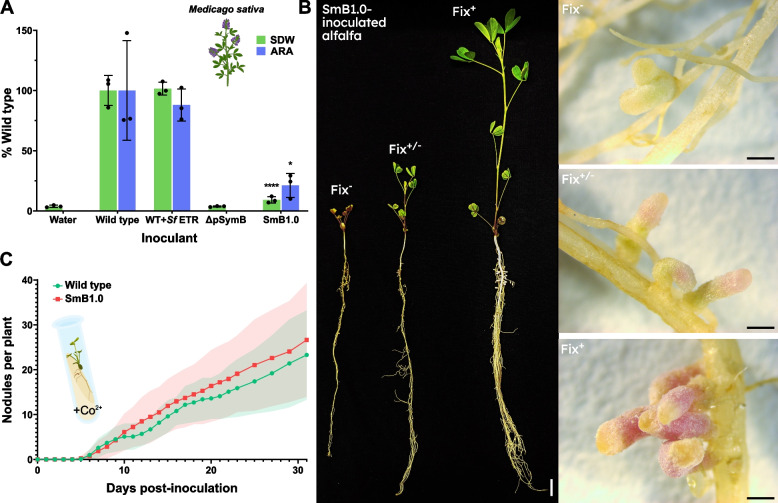
Symbiotic phenotypes of SmB1.0 with alfalfa. **A** Shoot dry weight of alfalfa inoculated with SmB1.0 was significantly reduced relative to plants inoculated with the wild type 35 days post inoculation (green bars). Nodules from SmB1.0-inoculated plants similarly reduce acetylene to ethylene far less efficiently than wild type nodules (blue bars) as determined by nmol ethylene/h/plant. The presence of the *S. fredii* ETR region does not appear to have a deleterious effect. **B** Variation in plant phenotype for alfalfa inoculated with SmB1.0. Within the same pot, plants ranged from Fix^−^ with small, white nodules, to strongly Fix.^+^ with nodules indistinguishable from the wild type. **C** Kinetics of nodule formation by SmB1.0 on alfalfa seedlings relative to wild type on Jensen’s agar slants with cobalt. Data is the average of 20 plants each. Shaded regions indicate standard deviation. Nodules emerged at a similar rate to wild type indicating SmB1.0 is sufficient for robust nodulation. Photograph scale bars represent 1 cm for the plants and 2 mm for the nodules. Shoot dry weight and acetylene reduction assays were assessed using one-way ANOVA followed by Dunnett’s multiple comparison test relative to the wild type. *p < 0.05, ****p < 0.0001

The symbiotic phenotype of *S. meliloti* strains were determined following the inoculation of *M. sativa* (alfalfa) seedlings and growth of the resulting plants in vermiculite/quartz sand with a plant nutrient solution lacking fixed nitrogen. Following 30–40 days of growth, uninoculated plants were chlorotic, stunted in growth, and lacked root nodules. In contrast, plants inoculated with wild-type *S. meliloti* grew vigorously with green leaves and roots carrying many pink root nodules, as is typical for *S. meliloti* strains that form an efficient N_2_-fixing symbiosis (Fix^+^). Alfalfa inoculated with the minSymB1.0 strain, SmB1.0 (RmP4256), exhibited a high degree of variation in plant size and nodule morphology, and this was reflected in markedly reduced average shoot dry weights (SDW) and reduced rates of N_2_-fixation (acetylene reduction assay, ARA) relative to wild type (Fig. 2A). The unusual and striking phenotypic variation in SmB1.0-inoculated alfalfa included plants that were short and chlorotic with white nodules, to some tall, healthy green plants with pink nodules akin to wild type (Fig. 2B). This variation was observed even among five to six plants growing within the same pot. The possibility that the variation resulted from contamination with strains that carried wild-type pSymB was investigated and eliminated as bacteria isolated from the pink nodules had the expected minSymB1.0 genotype (deduced by PCR analysis of pSymB loci) and Nm^R^ marker. In view of the variable, often reduced SNF phenotype of alfalfa inoculated with SmB1.0, we examined the kinetics of nodule formation to assess if the defect was nodulation related and found the kinetics of nodule formation being comparable to wild type (Fig. 2C). We added cobalt to the Jensen’s agar as minSymB1.0 omits the pSymB-encoded *cbtJKL* transporter of cobalt required at trace concentrations and Jensen’s does not include any cobalt supplementation [47, 48]. Without this cobalt supplementation, we observed a reduction in the proportion of SmB1.0-nodulated plants (Additional file 3: Fig. S2A) and the rate of nodule formation (Additional file 3: Fig. S2B). It therefore appears that the symbiotic impairment of minSymB1.0 is not due to delays in nodule formation and instead reflects an impairment at a more advanced stage of the symbiosis.

### The variation in the symbiotic phenotype of minSymB1.0 is not due to suppressor mutations in *S. meliloti*

**Fig. 3:**
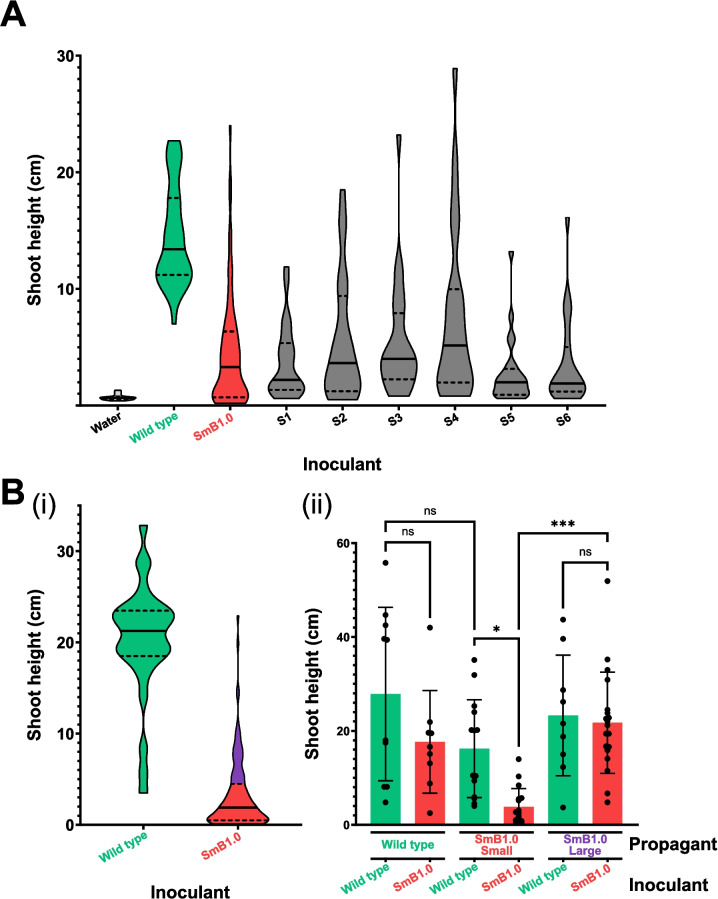
Plant height variation of alfalfa inoculated with SmB1.0 is due to variability of the plant, not a suppressor mutation in *S. meliloti*.** A** Violin plot showing shoot height distribution of individual alfalfa plants inoculated with wild type (*n* = 35) or SmB1.0 (*n* = 73) after 35 days. Re-inoculation of fresh alfalfa seedlings with isolates from the six tallest SmB1.0-inoculated plants, labelled S1-S6 (*n* = 24 for each), exhibited identical variation in plant heights as the original SmB1.0 inoculant. **B** (i) Violin plot showing initial shoot height distribution of individual alfalfa plants inoculated with wild type (*n* = 30) or SmB1.0 (*n* = 131) after 40 days. The upper quartile of SmB1.0-inoculated shoots is coloured purple. (ii) Propagants from wild type-, upper quartile of SmB1.0-, or lower three quartiles of SmB1.0-inoculated alfalfa upon re-inoculation with either wild type or SmB1.0. Tall and short plants from the initial round of SmB1.0 inoculation maintained their height phenotypes when re-inoculated with SmB1.0. Mean shoot height differences were assessed using one-way ANOVA followed by a Tukey multiple comparison test. *p < 0.05, ***p < 0.001

While the extremely variable phenotype of the minSymB1.0 strain is unusual, it was possible that this resulted from second-site suppressor mutation(s) arising in the SmB1.0 genome, as such mutations have been observed previously when plants were inoculated with certain Fix^−^ strains [49, 50]. To investigate this possibility, alfalfa seedlings were inoculated with either wild type (Rm2011) or SmB1.0, and the variation in plant heights following 35 days of growth was determined (Fig. 3A). As expected, several SmB1.0-inoculated plants were tall and green relative to the bulk of the plants that were small and chlorotic. Bacteria were isolated and streak-purified from the pink nodules of the six tallest plants and when tested, were Nm^R^ and unable to utilize galactitol as a sole carbon source like SmB1.0. The symbiotic phenotypes of these six purified isolates (S1-S6) were determined via the inoculation of alfalfa seedlings, and these showed variable phenotypes and were indistinguishable from the original SmB1.0 strain (Fig. 3A). We conclude that possible suppressor mutations in the minSymB1.0 strain were not responsible for the variable symbiotic phenotype.

*M. sativa* ssp. *sativa*, the typical cultivated alfalfa, is autotetraploid [51]. Populations are known to suffer from severe inbreeding depression which precludes inbred line formation, resulting in cultivars that are genetically mixed populations from crossing several different genotypes [52, 53]. It is thus possible that the observed variation in the minSymB1.0 symbiotic phenotype reflects the alfalfa genotypes in the cultivar population (i.e., SmB1.0 is robustly Fix^+^ for a sub-set of the population). To investigate this possibility, wild type- and SmB1.0-inoculated alfalfa plants were grown for 40 days to generate the same variation as before in plant heights (Fig. 3Bi). Cuttings from these plants were then clonally propagated (as outlined schematically in Additional file 4: Fig. S3 and described in Methods), re-inoculated with either wild type or SmB1.0, and grown in nitrogen-limiting conditions as usual. *S. meliloti* existing as endophytes appeared to be carried over in the cuttings and often were observed to nodulate without further need for additional inoculation, as might be expected [54]. Wild type-inoculated propagants had tall shoot heights regardless of whether they were re-inoculated with wild type or SmB1.0, likely due to the presence of endophytic *S. meliloti* from the original inoculum (Fig. 3Bii). However, when propagants from the lowest three quartiles of shoot heights that were inoculated initially with SmB1.0 were re-inoculated with SmB1.0, the resultant plants remained significantly shorter than when the same propagants received a wild-type re-inoculum (Fig. 3Bii). In such cases, a wild-type inoculum can over-ride the effects of a reduced SNF strain that may be present as an endophyte in the cuttings. Moreover, when propagants from the upper quartile of shoot heights inoculated initially with SmB1.0 (Fig. 3Bii, purple) were re-inoculated with SmB1.0, the resultant plants remained tall and were indistinguishable in height from propagants that received wild type (Fig. 3Bii). These data conferred strong support that SmB1.0 is robustly Fix^+^ on a sub-set of alfalfa plants that we presume have a particular genotype.

We also assessed the symbiotic performance of *S. meliloti* SmB1.0 on different seed stocks following the inoculation of fifteen alfalfa lines from four subspecies: *M. sativa* L. nothosubsp. *varia* (Martyn) Arcang., *M. sativa* L. subsp. *falcata* (L.) Arcang., *M. sativa* L. subsp. *caerulea* (Less. ex Ledeb.) Schmalh., and *M. sativa* L. subsp. *sativa* (Additional file 5: Table S2). No drastic deviations from the plant growth pattern we observed for SmB1.0 on *M. sativa* L. subsp. *sativa* cv. Iroquois., with most of the plants being small apart from the occasional tall plant.

### Nodules induced by SmB1.0 exhibit arrested development and senesce prematurely

**Fig. 4:**
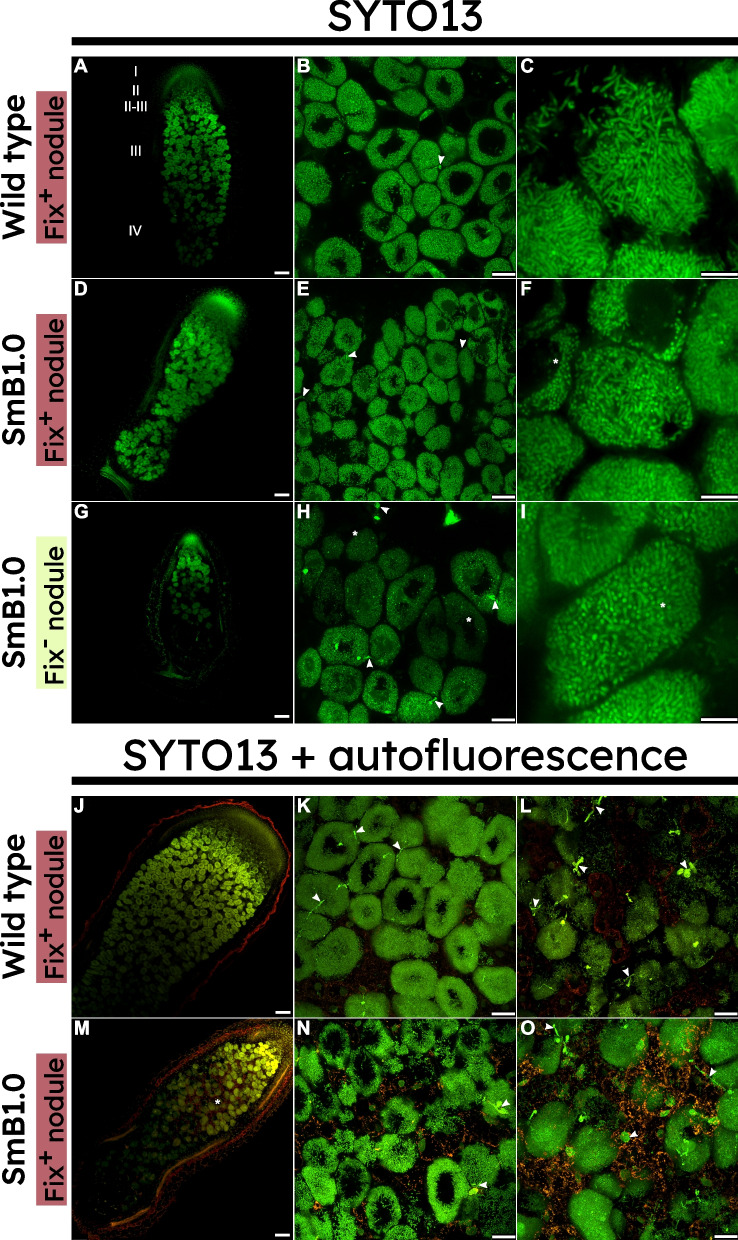
Laser scanning confocal microscopic analysis of alfalfa nodule structure and bacteroid morphology for SmB1.0-induced nodules 4 weeks post-inoculation show variable developmental and exhibit a higher degree of autofluorescence.** A** Wild-type nodules showed typical development with meristematic (I), infection (II), inter-(II-III), nitrogen-fixing (III), and senescent (IV) zones. **B,C** Infected cells of the nitrogen-fixing zone for wild-type nodules showing highly developed symbiosomes. **D**, **G** SmB1.0 nodules showing highly variable degrees of nodule development, occasionally developing normally (**D**) but more frequently arresting where the nitrogen-fixing zone should be (**G**). **E,F,H,I** Plant cells infected with SmB1.0 showing different degrees of bacteroid elongation that is less than for wild-type *S. meliloti*. Nodules were fixed and stained with the DNA-binding dye SYTO13. Arrowheads point to examples of bacteria in infection threads. Asterisks highlight poorly elongated bacteroids. **J,K** Wild-type nodules exhibited little overall proximal autofluorescence in nitrogen-fixing zone. **L** Wild-type nodules presented enhanced autofluorescent speckles in the senescent zone at the base of the nodule. **M–O** SmB1.0 nodules showing proximal autofluorescence (asterisk) and significant autofluorescence in the proximal portion of the nodule. Nodules were fixed and stained with the DNA-binding dye SYTO13 and autofluorescence was captured in the 600–700-nm range. Arrowheads point to examples of bacteria in infection threads. Asterisks highlight regions of pronounced autofluorescence. Images are representative of a minimum of five independent nodules examined. Scale bars: **A, D, G, J, M** 100 μm; **B, E, H, K–L, N–O** 25 μm; **C, F, I** 10 μm

We could readily distinguish white (Fix^−^), light-pink (Fix^+/−^), and pink (Fix^+^) nodules that formed on alfalfa inoculated with the minSymB1.0 strain (Fig. 2B). To gain insight into how far the nodule development progressed, particularly in the white (Fix^−^) nodules, nodule sections were examined via confocal laser scanning microscopy after staining with SYTO13. Mature wild-type nodules displayed the developmental gradation that is typical for indeterminate nodules, with a distal meristematic zone I, and infection, symbiotic, and senescent zones II, III, and IV, respectively [55] (Fig. 4A). Within the nitrogen-fixation zone (ZIII) of these nodules, bacteroids were elongated as expected (Fig. 4B-3). Alfalfa nodules induced by SmB1.0 displayed a level of variation expected given the other metrics examined. Tall plants had robustly Fix^+^ nodules similar in size to wild-type nodules (Fig. 4D), but with bacteroids appearing less elongated than those of wild-type nodules (Fig. 4E,F). In the case of small SmB1.0-inoculated alfalfa, the light-pink (Fix^+/−^) nodules displayed developmental arrest in the distal portion of the expected nitrogen-fixation zone (ZIII), which consequently contained few bacteroids (Fig. 4G). The most proximal bacteria of these nodules were visibly smaller and shorter, consistent with a lack of differentiation (Fig. 4H,I). Although not shown, spherical, white (Fix^−^) nodules of SmB1.0-inoculated plants still possessed visible bacteria inside, indicating development beyond the typically empty nodules induced by a strain lacking pSymB.

Autofluorescence is often indicative of a build-up of phenolic compounds associated with a plant defence response [56–58]. Autofluorescence speckles were detected at a wavelength of 600–700 nm in the proximal part (corresponding to ~ ZII-III in wild-type nodules) of Fix^+/−^ nodules induced by SmB1.0 (Fig. 4M–O). Contrastingly, these regions contained little autofluorescence in wild-type-induced nodules (Fig. 4J,K), with prominent autofluorescence only being observed in the senescent zone (ZIV) (Fig. 4L).

**Fig. 5:**
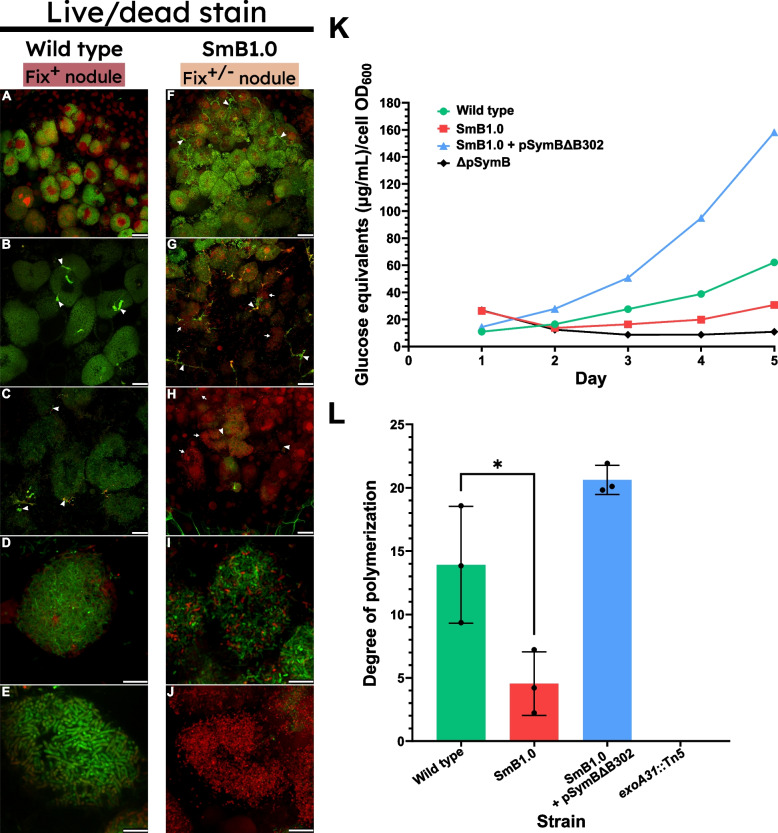
Live/dead staining of sections from Fix^+/^.^−^ alfalfa nodules induced by SmB1.0 reveals reduced bacteroid viability 4 weeks post-inoculation that coincides with drastically reduced amounts of high molecular weight succinoglycan.** A–E** Live/dead staining of wild-type nodules showing viable bacteroids in the infection zone (**A**), nitrogen-fixing zone (**B**), and near the base of the nodule (**C**). Viable, elongated bacteroids were observed (**D**, **E**) as expected. **F–J** Live/dead staining of SmB1.0 nodules showing viable bacteroids in the infection zone (**F**) but reduced bacteroid viability thereafter moving towards the proximal portion of the nodule where sporadic infection threads were observed (**G**). In some cases, the base of the nodule contained mostly dead bacteria (**H**). Variation in SmB1.0 morphology was again observed, with less-elongated, partially viable bacteroids being observed in some cases (**I**) but entirely dead, undifferentiated bacteria occupying the plant cell in others (**J**). Nodules were fresh and stained with a mixture of the DNA-binding dyes SYTO9 (green) and PI (red). Images are representative of a minimum of five independent nodules examined. Scale bars: **A–C**, **F–H** 25 μm; **D, E, I, J** 10 μm. Arrowheads point to examples of bacteria in infection threads. Full arrows point to examples of compromised bacteroid viability. **K,L** Succinoglycan production and molecular weight phenotypes of the SmB1.0 strain relative to the wild type. **K** Glucose equivalent concentration measured in μg/mL normalized to the optical density of the cell culture for the SmB1.0 and SmB1.0 + pSymBΔB302 strains compared to the wild type over a period of 5 days in GMS medium. The elevated ratio for Sm1.0 and ΔpSymB strains on day 1 is an artefact of those strains reduced growth rates. **L** Average degree of polymerization for the succinoglycan produced by SmB1.0 and SmB1.0 + pSymBΔB302 compared with wild type. No succinoglycan was detected for the *exoA31* mutant. Degree of polymerization was estimated by dividing the ratio of total hexose sugars to reducing sugar ends by eight (the number of hexose sugars per succinoglycan monomer). Assessments were made using one-way ANOVA followed by Dunnett’s multiple comparison test relative to the wild-type strain. *p < 0.05

Given the association of phenolic compounds and defence-like reactions with early nodule senescence [59], we investigated whether premature bacteroid death could be observed within the nodule using live/dead staining. For wild-type nodules, most bacterial cells stained with SYTO9 (green), indicative of intact cell membranes, regardless of whether they were within the distal portion (spanning ZI to interzone II-III between the infection and nitrogen-fixation zones; Fig. 5A), the nitrogen-fixation zone III (Fig. 5B), or near the base of the nodule (Fig. 5C). Fix^+/−^ nodules induced by SmB1.0 similarly illustrated intact bacteroid membranes in the infection zone of the nodule (Fig. 5F), but by the proximal part of the nodule exhibited increased propidium iodide (PI) staining (red), indicative of bacteroid membrane permeability (Fig. 5G) which often became more pronounced towards the base of the nodule (Fig. 5H). Infected plant cells from the proximal portion of wild-type nodules revealed viable, elongated bacteroids (Fig. 5D, E) whereas SmB1.0 nodules displayed a live/dead mixture of less-elongated bacteroids (Fig. 5I) or at the extreme, an array of red (dead) slightly elongated cells (Fig. 5J).

### Additional gene(s) on pSymB in conjunction with minSymB1.0 are required for a robust N_2_-fixing symbiosis with alfalfa

**Fig. 6:**
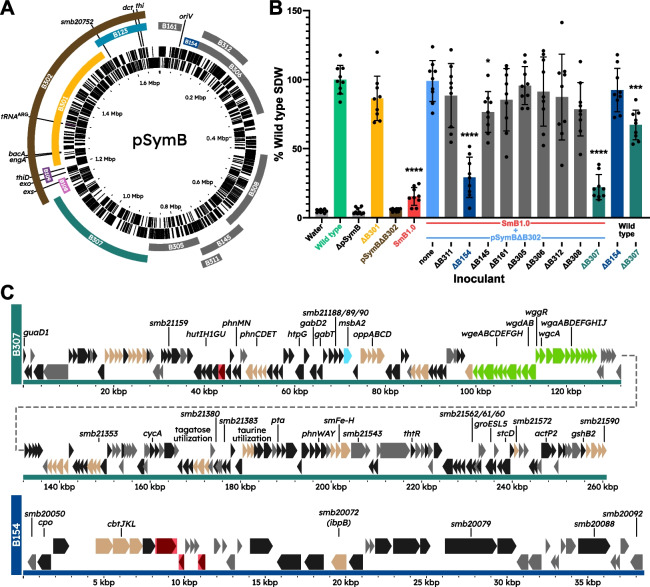
Map of *S. meliloti* pSymB showing the regions that were variously deleted in replicating pSymB constructs used to re-introduce into SmB1.0.** A** Full wild-type pSymB with B108, B109, B301, and B123 regions that are ultimately missing when B302 (brown) is removed. Shown outside of B302 are various deletions marked with gentamicin resistance that were independently transduced into pSymBΔB302 (except for B307 which was a FLP/*FRT*-mediated deletion). Relevant genes for efficient SNF are highlighted along with the essential *engA* and *tRNA*.^ARG^ genes within the B301 region. ***exo***: *exoA,B,F1,H,I,K-Q,T-Z*, ***exs***: *exsA-I*, ***dct***: *dctA,B,D*, ***thi***: *thiC,O,G,E*. **B** Shoot dry weight of alfalfa (*n* = 9) inoculated with SmB1.0 (red) was poor but was restored to wild-type levels (green) upon conjugal transfer of pSymBΔB302 (light blue). Independent conjugations of pSymBΔB302 carrying additional deletions into SmB1.0 were then performed. Horizontal bars on the *x*-axis legend indicate the presence of that construct in all encompassed strains. Data represent the mean of three independent experiments of three pots each. Most deletions had mild impacts on the ability of pSymBΔB302 to restore SmB1.0 SNF, apart from the constructs deleted in B154 (dark blue) and B307 (teal). **C** Detailed map of the regions and genes (represented as arrows) contained within the B307 and B154 regions. Genes encoding ABC transporters are shown as beige, while genes encoding proteins with predicted functions and hypothetical proteins are shown as black and grey, respectively. Genes highlighted in red indicate foreign insertion elements. Galactoglucan biosynthesis genes are lime green and previously implicated genes on the symbiosis are shown in cyan. Shoot dry weight assays were assessed using one-way ANOVA followed by Dunnett’s multiple comparison test relative to the wild-type strain. *p < 0.05, ***p < 0.001, ****p < 0.0001

The sub-optimal symbiotic phenotype of the *S. meliloti* minSymB1.0 strain suggested additional pSymB components are needed to generate a robust N_2_-fixing symbiosis with alfalfa plants. These could potentially be genes located within the 326-kb B301 region that are not complemented by a functional copy from the *S. fredii* ETR region (Fig. 6A). They could also be genes located within the 1162 kb pSymB region outside of the B108-B109-B301-B123 cluster (clockwise from the end of B123 to the start of B108 in Fig. 6A). This complete 1162-kb region was present on the pSymB deletion derivative, pSymBΔB302 (brown in Fig. 6A). Strikingly, when pSymBΔB302 was transferred to SmB1.0, the symbiotic phenotype of the resulting strain, RmP4370, was found to be indistinguishable from the wild type (light blue in Fig. 6B). This result suggested that the gene(s) needed to generate robust N_2_-fixing nodules that are missing from minSymB1.0 are present on the pSymBΔB302 replicon. Moreover, this result demonstrated that the 69-kb *S. fredii* ETR region with the *S. meliloti bacA* in minSymB1.0 is sufficient for complete symbiotic N_2_-fixation in the absence of the full 129-kb *S. meliloti* ETR and other genes in the 326-kb B301 region (Fig. 6A).

To identify the loci on pSymBΔB302 responsible for restoring robust N_2_-fixation to the minSymB1.0 strain, various sub-deletions of the pSymBΔB302 replicon (Fig. 6A) were analysed for their ability to support robust symbiotic N_2_-fixation upon transfer to SmB1.0 (Fig. 6B). Most of the deletions had minor impacts on the shoot dry weight of plants relative to plants inoculated with wild-type *S. meliloti*. However, a dramatic reduction in shoot dry weight was observed when pSymBΔB302 lacked the B154 region (i.e. ΔB154) (dark blue in Fig. 6A,B) or the B307 region (i.e. B307) (teal in Fig. 6A,B). In three independent experiments, plants inoculated with SmB1.0 carrying pSymBΔB302 with either ΔB154 or ΔB307 had on average 29 and 22%, respectively, of the shoot dry weight of plants inoculated with the wild type. Interestingly, the shoot dry weight of plants inoculated with an otherwise wild-type strain lacking the B154 or B307 regions (but containing the B302 region) were 92 and 67% of the wild type, respectively (Fig. 6B, two bars on the far right). Thus, loci within the B154 or B307 regions were more important for an efficient symbiosis when present in a minSymB1.0 versus an otherwise wild-type background. This suggests a negative epistasis between loci within the B301 region and loci within the B154 and B307 regions.

An examination of the gene content of the 38.5-kb B154 region (Fig. 6C) for loci that may be important for symbiotic N_2_-fixation revealed few obvious contenders, as the genes mostly encode hypothetical proteins or those with unknown function. Characterized genes include those encoding the CbtJKL cobalt ABC transport system and a solute-binding protein (SmB20072) induced by myo-inositol that is highly similar to IbpA (SmB20712) [47, 60].

The gene content of the 259.2-kb B307 region is similarly presented (Fig. 6C). Most notably present within this region are the *wga*,* wgcA*,* wggR*,* wgd*, and* wge* (formerly *exp*) genes required for the synthesis of galactoglucan (EPSII). Galactoglucan can support the symbiosis in place of succinoglycan; however, the strains employed here do not produce detectable amounts of EPSII owing to disruption of the *expR* (*smc03896*) regulatory gene by an ISRm1 element [61–63]. Also present are several clusters of genes (*phn*) involved in the transport and catabolism of phosphonates [50, 64]. The *stcD* gene, encoding an *N*-methylproline demethylase, is also within this region. StcD is involved in the catabolism of stachydrine, a compound exuded by *Medicago* seeds that enhances competitiveness for root colonization and the rate of nodulation, as well as activating NodD2 [65–68]. We note this region also contains the *msbA2* gene encoding an ABC exporter of a polysaccharide of unknown function whose removal is only deleterious when its upstream glycosyltransferase genes remain intact [69, 70].

### Altered succinoglycan production and composition in SmB1.0

A very large component of the minSymB1.0 set is the well-studied exopolysaccharide (EPS) gene cluster required for succinoglycan biosynthesis. Succinoglycan serves a critical role in the establishment of symbiosis via infection thread development as well as its more recently postulated late-stage symbiotic role in protection against nodule-specific cysteine-rich (NCR) peptides [71, 72]. Given the observed defects in nodule development and bacteroid viability, we analysed the minSymB1.0 strain on calcofluor-containing medium and found the strain to be very dim under UV relative to the wild-type strain, indicative of low EPS production (not shown). We subsequently investigated the production and composition of succinoglycan by SmB1.0 to assess if it differed from wild-type *S. meliloti* Rm2011. Over a period of 5 days of growth in GMS medium, SmB1.0 produced drastically less overall anthrone-positive material (i.e. carbohydrates) in its supernatant relative to the wild type but more than a strain that completely lacked pSymB (Fig. 5K). Complementation of minSymB1.0 with pSymBΔB302 (RmP4370) resulted in a substantial increase in anthrone-positive material over the wild type (Fig. 5K).

While a reduction in overall quantity is one factor of exopolysaccharide production, we also sought to determine if the minSymB1.0 set was producing an altered distribution of molecular weights of succinoglycan. Five-day-old cultures grown in GMS medium yielded CTAB-precipitable material that illustrated the SmB1.0 produced overall lower-molecular weight succinoglycan relative to the wild type (Fig. 5L). The EPS over-producing strain of minSymB1.0 carrying pSymBΔB302 had a succinoglycan composition of higher molecular weight chains (Fig. 5L). Given that a succinoglycan biosynthesis mutant (*exoA31*::Tn5) produced no significant anthrone-positive material, it was inferred that the detected sugar was almost entirely succinoglycan.

Overall, these data indicate that the minSymB1.0 set results in the production of less succinoglycan of a lower molecular weight. It should be cautioned here that the calculated molecular weights are relative and not indicative of the true distribution produced by the strain. This is because it is known that the methods of precipitation employed can favour the isolation of the higher molecular weight form of the polymer [73]. Strain SmB1.0 carrying pSymBΔB302ΔB307 (RmP4558) and an otherwise wild-type strain deleted in B307 (RmP2716) produced too little EPS to enable accurate analyses.

### A 673-kbp SymB replicon, minSymB2.0, is sufficient for effective N_2_-fixation with alfalfa

Evidently there are additional genetic loci needed for a consistently functional, minimized pSymB besides those included in minSymB1.0. Expectedly, inclusion of the B154 region with minSymB1.0 was insufficient to restore SNF appreciably over SmB1.0 (data not shown). The larger sizes of the B307 and B301 regions precluded their addition to the minSymB1.0 set as they could not be stably captured and transferred.

**Fig. 7:**
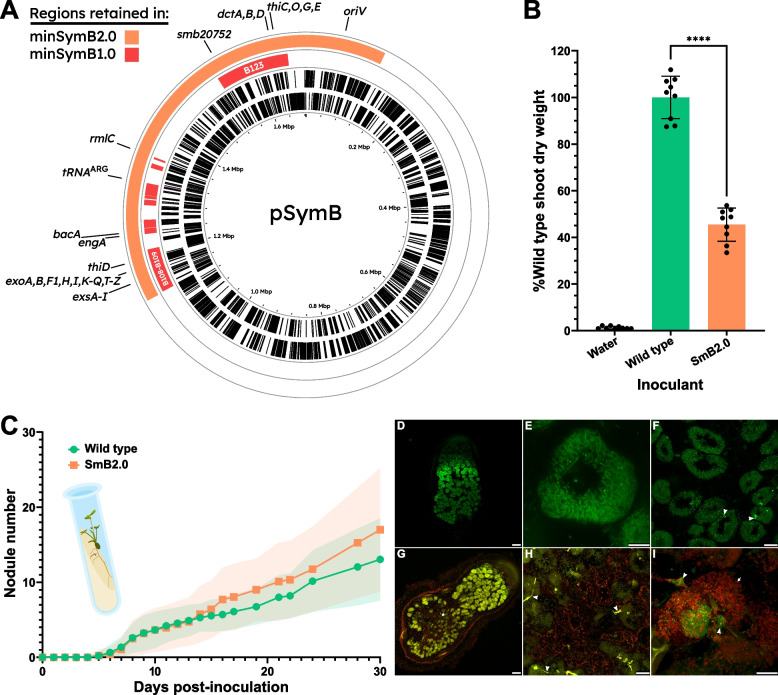
Genome of minSymB2.0 compared to minSymB1.0 and its subsequent symbiotic phenotypes.** A** Coloured tracks correspond to DNA that is present in the strain in question. From the outer to inner ring: minSymB2.0 (orange) and minSymB1.0 (red). **B** Shoot dry weight of alfalfa plants (*n* = 9) 35 days post-inoculation. Those inoculated with the minSymB2.0 strain (SmB2.0) were on average 50% of those inoculated with wild type. Data is the result of three independent experiments of three pots each. Shoot dry weight assays were assessed using one-way ANOVA followed by Dunnett’s multiple comparison test relative to the wild type strain. ****p < 0.0001. **C** Kinetics of nodulation of alfalfa seedlings with SmB2.0 showed no difference in the onset of nodulation relative to the wild type and only a slight increase in the number of nodules after the first 2 weeks. **D,E** Fixed SmB2.0 nodules with the DNA-binding dye SYTO13 display more developed nodules with more elongated bacteroids than was seen for SmB1.0 (**E**), although in some cases cells in the nitrogen-fixing zone contained poorly differentiated bacteroids (**F**). **G–H** The proximal portion (asterisk) of SmB2.0 nodules was often observed to strongly autofluoresce in the 600–700-nm range. **I** Live/dead staining of SmB2.0 nodules revealed in some cases plant cells occupied by mostly dead, poorly elongated bacteria that were associated with infection threads harbouring live bacteria. Nodules were fresh and stained with a mixture of the DNA binding dyes SYTO9 (green) and PI (red). Images are representative of a minimum of five independent nodules examined. Scale bars: **D, G** 100 μm; **F, H** 25 μm; **E, I** 10 μm. Arrowheads point to examples of bacteria in infection threads. Asterisks highlight regions of pronounced autofluorescence. Full arrows point to examples of compromised bacteroid viability

In view of the apparent epistasis between loci in the B301 region and loci in B154 and B307, we sought to employ another approach to derive a minimal symbiotic pSymB. We constructed a pSymB derivative in a wild-type (RmP110) background that retained the full B301 region (with complete *S. meliloti* ETR) but had the 1008-kb region from pSymB nt 122,109–1129757 removed. The resulting strain, SmB2.0, carries a 673-kb pSymB replicon whose content was designated minSymB2.0. This replicon includes the B108-B109-B301-B123 regions, as well as the B154 region, but not the B307 region. Alfalfa plants inoculated with SmB2.0 formed pink, Fix^+^ nodules and the shoot dry weight of the resultant plants was 40–50% of plants inoculated with wild-type *S. meliloti* (Fig. 7B). Assessment of the kinetics of nodule formation relative to the wild type suggested no significant impairment (Fig. 7C). Four-week-old nodules induced by SmB2.0 appeared more similar to the wild type than those induced by SmB1.0 previously (Fig. 7D). Bacteroids were fully elongated in the early portion of the N_2_-fixing zone (Fig. 7E); however, in the more proximal part of the nodule they appeared smaller and disordered (Fig. 7F). A subsequent analysis of 600–700-nm range autofluorescence revealed a significant amount of highly fluorescent speckles beginning in the middle of the nodule and spanning to the base (Fig. 7G,H). Live/dead staining revealed in some cases that live (green) bacteria were present in infection threads leading to plant cells filled with dead bacteria (red) (Fig. 7I). The 673-kb minSymB2.0 represents the smallest, moderately efficient Fix^+^ pSymB derivative that has been constructed.

### Symbiotic phenotypes of minimized pSymB strains are more severe on the alternate host *Melilotus officinalis* (yellow sweet clover)

The variation in the symbiotic phenotype of alfalfa plants inoculated with the SmB1.0 prompted us to examine the symbiotic phenotype on *Melilotus officinalis* as an alternative host. Curiously, the SmB1.0-inoculated *M. officinalis* plants carried small, white Fix^−^ nodules and the shoot dry weight of these plants did not differ appreciably from the un-inoculated control plants (Additional file 6: Fig. S4A). Furthermore, acetylene reduction measurements showed no nitrogenase activity from SmB1.0-induced *M. officinalis* nodules (data not shown). Microscopic examination of the SmB1.0-induced *M. officinalis* nodules revealed that few plant cells proximal to the infection zone II-III contained bacteroid-like cells (Additional file 6: Fig. S4E-J). This is consistent with an apparent arrested development and rapid senescence of bacteroids in SmB1.0-induced Fix^−^ nodules in contrast to the large symbiotic zone III produced by the wild type (Additional file 6: Fig. S4B-D). The transfer of pSymBΔB302 to SmB1.0 restored the symbiosis to wild-type levels, whereas *M. officinalis* plants inoculated with SmB1.0 carrying pSymBΔB302ΔB307 were Fix^−^ like SmB1.0 (Additional file 6: Fig. S4A). These data mirrored the effect of pSymBΔB302 and pSymBΔB302ΔB307 on the SmB1.0 symbiotic phenotype of alfalfa nodules. However, the deletion of either B154 or B307 regions in an otherwise wild-type pSymB background had a more severe reduction in shoot dry weight of inoculated *M. officinalis* than we observed with alfalfa (Additional file 6: Fig. S4A and Fig. 6B). The completely Fix^−^ phenotype of SmB1.0 carrying pSymBΔB302ΔB307 (RmP4558) seemed an attractive background into which a wild-type *S. meliloti* clone bank could be inserted to restore symbiosis with *M. offiinalis* [74]. However, no noticeably Fix^+^ plants were obtained after having examined > 6000 independent Tc^R^ transconjugants in which RmP4558 received pLAFR1 harbouring *S. meliloti* DNA. Our 673-kb pSymB present in SmB2.0 was Fix^+^ with *M. officinalis*, but the shoot dry weight of inoculated plants was reduced to 31% of the wild type.

## Discussion

We have outlined the process and progress made towards defining the minimal pSymB genome that allows for robust symbiotic N_2_-fixation with alfalfa. In previous work, three regions of pSymB (B108, B109, and B123) were shown to be essential for N_2_-fixation and numerous other regions also appeared important as their removal reduced the symbiotic phenotype with alfalfa [29]. To generate a minimal, symbiotic pSymB genome, the B108-B09 and B123 regions were combined and inserted into the *S. meliloti* chromosome of a pSymA^+^ ΔpSymB strain in which the smaller ETR region from *S. fredii* NGR234 (with the *S. meliloti bacA*) was chromosomally integrated. Inoculation of alfalfa with the resulting minSymB1.0 strain (SmB1.0) generated a surprising symbiotic phenotype with an unusually high degree of variation. Most plants formed white to light-pink nodules that fixed little nitrogen; however, some alfalfa plants grew vigorously and carried pink root nodules. Notably, similar variation in symbiotic outcome with alfalfa was observed when the *S. meliloti bacA* gene was replaced with the *bacA* of *S. fredii* [45].

Two lines of evidence suggest that the variation in the symbiotic phenotype observed in both studies is likely due to the variability in the genotype of the *M. sativa* cv. Iroquois seed stock. Firstly, in both studies, when bacteria from the pink nodules were re-inoculated onto alfalfa seedlings, the resulting plants exhibited the same variable phenotype as did the original strains. Secondly, when the healthy green plants obtained following inoculation with SmB1.0 were vegetatively propagated, they retained the Fix^+^ phenotype and the nodules on these plants carried SmB1.0. Nodulation kinetics and microscopic examination of the white and pink nodules from SmB1.0-inoculated plants demonstrated that the initiation of nodule formation and the release of bacteria from the infection thread proceeded normally but that most of the resulting nodules senesced prematurely. Whether this senescence is triggered by a plant defence response or metabolic defects in the bacteria, or a combination of these factors, is unclear. Our ability to identify and vegetatively propagate alfalfa plants that allow efficient SNF with SmB1.0 offers an avenue to identify the apparent genetic basis for this phenotype. Indeed, recent developments in alfalfa genome analysis have overcome the challenges associated with self-incompatibility and its tetraploid genome [75, 76]. In future experiments, crossing the large and small alfalfa phenotypes associated with SmB1.0 inoculation may elucidate the nature of the underlying genotype.

The finding that our minSymB1.0 strain forms some pink, Fix^+^ nodules suggests that minSymB1.0 carries the major determinants from pSymB that are necessary for symbiotic N_2_-fixation despite representing an 84.5% decrease in pSymB gene content. The minSymB1.0 content includes known important symbiotic loci: *exo*-*exs* loci encoding for succinoglycan biosynthesis as well as for genes involved in queuosine biosynthesis, *dct* loci for C_4_-dicarboxylic acid transport, s*mb20752* encoding a putative 3-hydroxyisobutyryl-CoA hydrolase, and *bacA* required for normal bacteroid development [77–82]. However, SmB1.0 clearly did not support robust SNF with alfalfa and formed exclusively Fix^−^ nodules on the alternate host *M. officinalis*. Consequently, subsequent work focused on identifying additional pSymB loci that would bolster the symbiotic proficiency of minSymB1.0. As noted from the previous deletion analysis [29], many loci outside of the B108-B109 and B123 regions appear to influence symbiotic effectiveness.

Importantly, the transfer of most of the pSymB replicon that is not present in minSymB1.0 (i.e. pSymBΔB302) to SmB1.0 restored a complete wild-type SNF phenotype. Two loci within pSymBΔB302—B154 and B307—were required for the complete restoration of SNF to SmB1.0. Intriguingly, otherwise wild-type *S. meliloti* in which the B307 or B154 regions were deleted were robustly Fix^+^ on alfalfa, whereas their deletion in pSymBΔB302 severely compromised SNF when introduced into SmB1.0. Critically, the symbiotic phenotype of *S. meliloti* with the ΔB301 deletion and the *S. fredii* ETR construct in the chromosome [ΩETR(*Sf*) Δ*bacA*(*Sf*)::*bacA*(*Sm*)] was indistinguishable from the wild type (RmP4234, yellow in Fig. 6A,B). This suggests that any symbiotic defect resulting from the loss of the *S. meliloti* ETR or other loci within the B301 region was fully compensated for by the presence of the *S. fredii* ETR on the chromosome. This is consistent with the finding that SmB1.0 carrying pSymBΔB302 forms fully functional Fix^+^ nodules. Thus, a major symbiotic defect is observed when pSymB lacks the DNA from the B154 or B307 regions in addition to the B301 region. Therefore, it appears that the B154 and B307 regions harbour uncharacterized symbiotic loci whose removal is cumulatively deleterious along with the removal of additional loci within B301 region that were not compensated by the *S. fredii* ETR genes. Our inability to restore the symbiosis of the SmB1.0 strain carrying pSymBΔB302ΔB307 on *M. officinalis* with a clone bank of wild-type *S. meliloti* DNA (average insert size = 23 kb), despite screening significantly more (~ 6 ×) transconjugants than would be expected to recover the phenotype if a single locus was responsible, suggests potentially multiple, distant genes within B307 may be involved [74]. The nature of these negative epistatic interactions appears complex and requires further investigation. An immediate possibility is that the loci contain gene(s) that are functionally redundant with respect to SNF to loci within either B154 or B307. Whether these gene(s) encode something needed directly by the bacterium or something exported for interaction with the plant remains to be determined. It is worth noting that *S. meliloti* does not possess a type III secretion system used by other rhizobia to deliver effectors, nor does its type IV secretion system seem to be needed for SNF [83, 84].

Observations of SmB1.0-induced nodules revealed the release of viable bacteria that fail to fully elongate and ultimately senesce prematurely. This is a similar outcome to what is seen in non-functional nodules of *M. truncatula* mutants with elevated defence or premature senescence phenotypes [85–87]. Coupled with the enhanced autofluorescence in infected plant cells, it appears SmB1.0 is succumbing to the plant’s defence response. Whether this is due to the diminished ability to suppress the host’s defences or enhanced susceptibility to them would require additional study. These observations prompted us to examine the exopolysaccharide production by these strains as similar observations have been made for succinoglycan biosynthesis mutants of *S. meliloti* [88]. SmB1.0 produced a decreased amount of more predominantly low molecular weight (LMW) succinoglycan than the wild type. The addition of pSymBΔB302 resulted in more succinoglycan than the wild type that skewed to that of a higher molecular weight (HMW) form. An immediate reasoning for this increased abundance may stem from the removal of the *emmABC* gene cluster (located within B301 and not compensated by the *S. fredii* ETR), for which mutations have previously been associated with increased succinoglycan production but still effective symbioses with alfalfa [89, 90]. However, these studies do not assess the relative molecular weight of the exopolysaccharide produced by these mutants. Curiously, SmB1.0, which also lacks the B301 region containing *emmABC*, does not overproduce succinoglycan in the same manner. The production of more succinoglycan in the HMW form can also be potentially explained by the removal of the *prsDE* genes which encode a type I secretion system that exports the ExsH glycanase that cleaves the HMW form of succinoglycan [91, 92]. This would suggest the existence of additional loci elsewhere on pSymB whose removal results in a decreased production of LMW-skewed succinoglycan that is dominant to the removal of the gene(s) in the B301 region that cause an increase of the HMW form.

Small amounts of LMW EPS are known to be sufficient for successful invasion of the plant host [93]. Thus, a more likely explanation for the symbiotic defects of SmB1.0 may lie in some of the emerging additional functions of succinoglycan in the symbiosis. HMW succinoglycan has been hypothesized to play an important role in survival against NCR peptides that govern terminal bacteroid differentiation [71]. SmB1.0’s reduced bacteroid viability may reflect the strain’s compromised production of HMW succinoglycan in later stages of the symbiosis rendering the bacteroids overly sensitive to NCR peptides. The eventual loss of viability and premature senescence of nodules despite obvious developmental progression is reminiscent of *S. meliloti* carrying the incongruent *S. fredii bacA* gene in lieu of its own on alfalfa [45]. BacA is essential for the *S. meliloti*-*Medicago* symbiosis, being required for proper bacteroid elongation and prevention of senescence [79, 94]. BacA appears to uptake certain NCR peptides that control bacteroid differentiation and drive nitrogenase activity [95–97]. While its key role in planta is localization of NCR peptides to the cytoplasm, it has the ancillary effect of protecting the *S. meliloti* bacteroids from the bactericidal effects of the NCR peptides [94]. It is possible then that SmB1.0-induced bacteroids are compromised in the ability to resist host NCR peptides in a similar manner to *bacA* mutants and hybrid constructs, possibly due to compromised succinoglycan output/composition or other factors. Despite the presence of over 600 expressed NCR peptides in *M. truncatula*, the deletion of single *NCR* genes is sufficient to severely disrupt the symbiosis [98–103]. Furthermore, it has been shown that for *M. truncatula*, genotypic NCR peptide differences negatively impact SNF in a strain-specific manner [104–106]. It is thus tempting to speculate that the phenotypic variation observed in SmB1.0-inoculated alfalfa is due to variation in the plants’ NCR peptide profiles. Despite also being highly self-incompatible, the diploid nature of *M. officinalis* would be expected to confer a lower degree of heterozygosity in the seed population [107, 108]. Coupled with the different genetic background, we hypothesize that the NCR peptide profile of *M. officinalis* is such that SmB1.0 exhibits a consistent failure to sustain differentiated bacteroids.

At the outset of this work, the goal of generating a minimal pSymB symbiotic genome appeared remarkably challenging. It has been observed that the singular removal (deletion) of numerous regions encompassing the entirety of pSymB results in slight reductions to the SNF phenotype in nearly all cases [29]. This differed to what was observed for pSymA, where most deletions had no effect on the SNF phenotype. Hence, the generation of a minimal pSymA genome was very successful in establishing that only 75 kb of the 1354 kb pSymA genome was sufficient to nodulate alfalfa with near wild type symbiotic efficiency [29, 37].

## Conclusion

Despite the inherent complexities of the 1683 kb pSymB genome, we have established that a 60% minimized set of 673 kb (minSymB2.0) is sufficient to generate alfalfa nodules with 45% of the symbiotic efficiency of the wild type. Minimizing pSymB further appears to be a promising endeavour for uncovering new, symbiotically relevant loci. This is especially true for those whose removal appears to only be severely deleterious in the presence of the removal of other, often distant regions on the chromid. Moreover, such approaches begin to unravel the complex nature of quasi-essential genes for the symbiosis, whose cumulative removal becomes too great of a burden for efficient SNF to occur. The minSymB2.0 strain established here is a favourable starting point for continued minimization, as it is already missing over 1 Mb of pSymB yet is consistently forming an effective symbiosis with alfalfa. Expanding upon the deletion tools utilized here, coupled with the yeast-based homologous assembly workflows employed previously [37], we hope that continued minimization will facilitate identification of these unknown symbiotic players.

## Methods

### Bacterial strains and growth conditions

*Escherichia coli* was typically grown in lysogeny broth (LB) (Lennox) at 37 °C. *S. meliloti* was grown in LB supplemented with 2.5 mM MgSO_4_ and 2.5 mM CaCl_2_ (LB_mc_) at 30 °C. Strains lacking pSymB or regions containing the *cbtJKL* genes encoding an important cobalt transporter were grown in LB_mc_ supplemented with 38 μM FeCl_3_ and 2 μM CoCl_2_ [47]. For the recovery of *E. coli* transformant cells after electroporation, super optimal broth with 20 mM glucose (SOC) was used. Experiments involving minimal media with a sole carbon source utilized M9 minimal media (1 × M9 salts, 1 mM MgSO_4_, 0.25 mM CaCl_2_) to which 10 ng/mL CoCl_2_, 1 μg/mL thiamine hydrochloride, 1 μg/mL biotin, and either 10 mM of glucose, sucrose, or taurine were added. The final concentrations of antibiotics used in solid agar media were 200 μg/mL streptomycin (Sm), 100 μg/mL spectinomycin (Sp), 200 μg/mL for neomycin (Nm), 60 and 10 μg/mL gentamicin (Gm) for *S. meliloti* and *E. coli* respectively, 5 μg/mL tetracycline (Tc), 5 μg/mL chloramphenicol (Cm), 50 μg/mL rifampicin (Rif), and 25 μg/mL kanamycin (Km). All concentrations were halved for growth in liquid media.

Bacterial strains and plasmids utilized in this study are listed in Additional file 7: Table S3. Primers utilized are listed in Additional file 8: Table S4.

### Genetic manipulations

Triparental conjugal transfer of plasmids from *E. coli* DH5α to *S. meliloti* was performed using a third helper plasmid, pRK600, in *E. coli* strain MT616 as described previously [109–111]. Mating mixtures were spotted directly on LB or LB_mc_ and grown overnight at 30 °C. Transductions of marked deletions from one *S. meliloti* strain to another were performed with the lytic phage ΦM12 using the protocol of Finan et al. [112]. Routine enzymatic digestions and ligations were performed according to the manufacturer’s instructions (New England Biolabs, Ipswich, Massachusetts). Singular, large deletions of the pSymB chromid were made utilizing the pTH1937/pTH1522 Flp-mediated system described previously [113].

### Symbiotic assays

Plant growth experiments utilizing *M. sativa* cv. Iroquois were performed in Leonard jar assemblies made from Magenta boxes as described previously [29, 37] with the exception that plants were grown for 28–35 days. Acetylene reduction assays of nitrogenase activity were performed with an HP6890 gas chromatograph (Agilent Technologies, Santa Clara, California) as described previously [114, 115] by taking measurements at 6-min intervals for 18 min. Nodule kinetics were assessed by counting the formation of nodules every 1–2 days on individual seedlings grown on 1% Jensen’s medium [48] agar slopes in glass tubes that received 100 μL of ~ 10^7^ *S. meliloti* cells/mL in sterile water. The addition of 2 μM cobalt to Jensen’s agar slants was required for nodulation kinetics assays involving strains that lacked *cbtJKL*. We note that while all symbiotic assays performed in Leonard jars with sand/vermiculite supplemented with Jensen’s medium lacked this additional cobalt, the addition of 2 μM cobalt to Jensen’s medium conferred no benefit on nodule metrics or shoot dry weight of inoculated alfalfa in constructs that are missing the *cbtJKL* operon (Additional file 9: Fig. S5). This is unsurprising given cobalt is present in abundance in vermiculite, albeit with unknown bioavailability [116].

### Vegetative propagation of alfalfa

*S. meliloti*-inoculated alfalfa plants to be asexually propagated were grown for 40 days and then cut-back above the bottom node. The media in the lower Leonard jar compartment was replaced with 200 mL of fresh Jensen’s medium supplemented with 10 mM KNO_3_. Cut-back plants were then grown for a week in a 7-inch propagation dome (Mondi Products, Vancouver, British Columbia) and grown for another 2 weeks with the dome removed. The cut-back and regrowth cycle was repeated a minimum of three times to ensure homogeneity of the plant population. Cuttings 5–10 cm in in length were taken from shoots of the re-grown alfalfa plants to include 2–3 petioles and shoots were removed. The lowest node was dipped into sterile water and then into 0.1% indole-3-butyric acid (IBA) rooting powder (PRO-MIX STIM-ROOT, Premier Tech, Rivière-du-Loup, Quebec) and buried into sterilized 1:1 w/w vermiculite sand in a Leonard jar assembly with a cotton wick that contained 250 mL of sterile Jensen’s medium lacking nitrogen. Propagated plants (propagants) were placed in propagation domes and allowed 2 weeks to root under the same growth conditions as for the symbiotic assays. Propagants were inoculated with *S. meliloti* after this time and grown for a further 35 days with the dome lid removed. This process is visually summarized in Additional file 4: Fig. S3.

### Microscopy

Mature nodules were harvested into 80 mM PIPES (pH = 7.0). Nodules were fixed in 4% formaldehyde in 80 mM PIPES and vacuum infiltrated 3 × for 30 s each, venting in-between, followed by 30 min incubation at room temperature. Nodules were thoroughly rinsed in 80 mM PIPES 3 × 5 min each with shaking, before being patted dry and encapsulated in 6% (w/v) low melting point agarose in phosphate-buffered saline (PBS) (pH = 7.4) and cut into blocks. Agarose blocks were mounted with cyanoacrylate adhesive onto the specimen plate and longitudinally sectioned into 70-μm thin sections using a Leica vibratome VT1000 S vibrating blade microtome (Leica Biosystems, Nussloch, Germany) with one half of a double-edged razor blade. Sections both fixed and not were harvested into ice-cold PBS and stored overnight at 4 °C prior to staining. Fixed nodule sections were stained in the dark with 1 µL/mL SYTO13 (Invitrogen, Waltham, Massachusetts) stain in PBS (5 µM final) with shaking for 15 min [117]. For live/dead staining, non-fixed, fresh nodule sections were stained in the dark for 20 min with live/dead staining solution [5 µM SYTO9 and 30 µM propodium iodide (PI) in 50 mM Tris–HCl, pH = 7.0] (LIVE/DEAD *Bac*Light bacterial viability kit, Invitrogen, Waltham, Massachusetts). Sections were removed from the staining solution and mounted in 80% (v/v) glycerol in PBS using a No. 1 cover glass system. Images were then acquired at 1024 × 1024 pixels resolution utilizing a Leica SP5 confocal laser scanning microscope using a Leica × 10 HCX PL APO CS (NA 0.40), × 20 HCX PL APO CS (NA 0.70), or × 63 HCX PL APO CS (NA 1.40) objective lens (Leica Microsystems, Wetzlar, Germany). Images of SYTO13 fluorescence or autofluorescence were acquired using a 488-nm laser line with a 493–531-nm emission channel (SYTO13, green) and 600–700-nm emission channel (autofluorescence, red). Images for live/dead staining were acquired using a 488-nm laser line with a 494–521-nm emission channel (SYTO9, green) and a 598–665 nm emission channel (PI, red). Images represent maximum 2D-sections or 3D-projections of acquired Z-stacks utilizing the accompanying LAS AF software.

### Genome sequencing and analysis

Genomic DNA from *S. meliloti* strains SmB1.0 (RmP4256), SmB1.0 + pSymBΔB302 (RmP4370) and its derivatives with ΔB154 or ΔB307 (RmP4551 and RmP4558), and SmB2.0 (RmP3560) were sequenced (Illumina HiSeq) at the McMaster Genomics Facility at McMaster University. Reads were trimmed using BBDuk and aligned to a map of the expected in silico genome sequence with Geneious Prime software (Dotmatics, Boston, Massachusetts) (mapper: Geneious; sensitivity: medium sensitivity/fast; fine-tuning: up to five iterations; reads not trimmed). An MMseqs2 [118] reciprocal best hits (RBH) analysis was used to identify orthologous genes between the *S. fredii* and *S. meliloti* ETR regions.

### Isolation and quantification of EPS

Qualitative assessment of EPS production was performed by growing the strains on LB_mc_ containing 0.02% calcofluor white (Fluorescent brightener 28) and viewing under UV light [119].

For quantitative assessment of EPS production, strains to be analysed were grown overnight at 30 °C in 3 mL of LB_mc_ before being centrifuged and washed twice with 0.85% (w/v) NaCl and re-suspended in GMS medium as per Zevenhuizen & van Neerven [120] with 55 mM D-mannitol. Cells were sub-cultured in 50 mL GMS to an OD_600_ = 0.01 and grown for 5 days at 30 °C with rotation (230 rpm). Cultures were centrifuged for 10 min at 20,000 × *g* to pellet cells and the clear supernatant was removed to a fresh tube. Cell pellets were flash frozen and assayed for total protein. To precipitate acidic exopolysaccharides from culture supernatants, 0.3 volumes of 1% cetyltrimethylammonium bromide (CTAB) was added, followed by an additional centrifugation for 10 min at 20,000 × *g* to pellet precipitate [121, 122]. Supernatant was discarded and the pellet re-dissolved in 10% NaCl with shaking at 37 °C for 1–24 h (depending on EPS quantity), followed by re-precipitation with 3 volumes of ice-cold acetone [123–125]. Precipitate was centrifuged for 20 min at 20,000 × *g* and the supernatant was discarded. The pellet was air dried and re-suspended in sterile deionized water.

Total carbohydrate content was assayed using the anthrone-H_2_SO_4_ method [126] with modifications [127], utilizing a glucose standard curve. This was adapted to a 96-well microplate format in which 100 µL of ice-cold 0.1% (w/v) anthrone dissolved in 95% (v/v) sulphuric acid was dispensed into each well of a plate chilled on ice. Fifty microlitres of sample or standard was carefully layered on top and the plate was mixed by shaking. The plate was then incubated on a pre-heated aluminum block in a 100 °C oven for 30 min before being cooled on ice for 5 min and left standing at room temperature while covered from light for another 20 min. Absorbance of each well was then measured at 620 nm.

Total reducing sugar content was quantified using the copper(II)-neocuproine assay adapted to a 96-well microplate scale based upon Bener et al. [128] with the modification of utilizing neocuproine-HCl dissolved in water and glycine as the copper chelating agent (340 mM) as per Dygert et al. [129], with a glucose standard. The ratio of total sugar content (anthrone-H_2_SO_4_ assay) to reducing sugar content (Copper-neocuproine assay) was used to estimate the molecular weight of succinoglycan in the sample as each molecule has a single reducing end regardless of polymer length [130]. This value was divided by eight to estimate the degree of polymerization for the sample as a single succinoglycan molecule possesses 8 sugar residues (7 glucose and 1 galactose) [131].

### Construction of *S. meliloti* strain SmB1.0

A schematic representation of the construction of SmB1.0 is provided in Additional file 2: Fig. S1.

To delete the B301 region, primer pair HR_1_Fwd/HR_1_Rev was used to amplify a 663-bp region of homology from pSymB (nt 1,203,758–1204420) at the right flank of the B109 region, and primer pair HR_2_Fwd/HR_2_Rev was used to amplify a 491-bp region of homology from pSymB (nt 1,530,068–1530558) at the left flank of the B123 region (Fig. 1A) [113]. The primer pairs (Additional file 8: Table S4) included mutant *loxP* sites *lox71* and *lox66* in the reverse primer of HR_1 and the forward primer or HR_2, respectively [132]. These two fragments were digested with BamHI and KpnI, respectively, and cloned into pJQ200SK (containing *sacB *[133]) and pTH1945, respectively, to yield pTH3244 and pTH3242. The pTH3242 construct was subsequently conjugated into *S. meliloti* RmP3957 (containing the *S. fredii* ETR region on the chromosome with the *S. meliloti bacA* gene) [45], whereby it recombined into pSymB via single homologous recombination. Transconjugants were selected for on LB_mc_ with Sm and Nm, forming RmP4224. A second conjugal transfer of pTH3244 into *S. meliloti* RmP4224 was performed and selected for on LB_mc_ with Sm and Gm to form RmP4227 (Fig. S1A1). In order to recombine the two *loxP* mutant sites now located on pSymB in RmP4227, pJG468, a promoterless *cre* integration vector that contains 451 bp of homology to the end of the *tauC* gene of the taurine inducible operon on pSymB [134] was integrated into wild-type pSymB and subsequently transduced into *S. meliloti* RmP4227 to ensure *cre* was correctly located under the control of the taurine-inducible promoter P_tauA_ (Fig. S1A2). Overnight cultures of this strain were grown in LB_mc_ supplemented with 10 mM taurine to induce expression of the Cre recombinase (Fig. S1A3). Cells were diluted and plated on LB_mc_ with 5% sucrose as counterselection against strains in which Cre did not recombine the two *loxP* sites. The taurine operon in *S. meliloti* was proposed to include *tauX* and *tauY* encoding the small and large subunits of a taurine dehydrogenase downstream of *tauABC* [135]. Therefore, pJG468 integration should block the ability of RmP4233 to utilize taurine. The resulting deletion strain was successfully cured of the pJG468 integrant by selecting for the ability to grow on M9 minimal media with 10 mM taurine as the sole carbon source, which was named pSymBΔB301 (RmP4234).

To facilitate excision of the now adjacent B108/B109/B123 regions in pSymBΔB301, the Flp-mediated recombination of *FRT* sites previously described by Milunovic et al. [113] was used. An existing plasmid from the *S. meliloti* fusion library [136] (Cowie et al., 2006), pFL916, already contains a region of homology to the outer right flank of B123 and possesses an *FRT* site. However, the pTH1937 derivative was recreated with the region of homology to the left flank of B108 downstream of the *FRT* site. This was critical to ensure that once recombined via Flp, the excised plasmid contains a selectable marker and origin of transfer to enable stable capture in *E. coli*. Primers 1938 F and 1938R were used to amplify pSymB nt 1,129,758–1,131,168 from RmP110 gDNA, which was then cloned into pTH1937 via KpnI/HindIII to form pTH3243. Conjugation and subsequent selection for single homologous crossover integrants of plasmids pTH3243 (Nm^R^) followed by pFL916 (Gm^R^) into RmP4234 formed RmP4241 (Fig. S1A4). To enable Flp-mediated recombination of the two *FRT* sites contained within the pTH3243 and pFL916 integrants, the unstable Tc^R^ plasmid pTH2505 [137] was conjugated into RmP4241, yielding RmP4242 (Fig. S1B1). A triparental mating was set up between RmP4242, MT616, and Rif-resistant *E. coli* DH5αR [138] on LB_mc_ containing 2.5 mM protocatechuic acid (PCA) to induce the expression of *flp* on pTH2505 (Fig. S1B2). Flp-mediated recombination of the *FRT* sites on pSymB of RmP4242 yielded a large plasmid containing an *oriT*, selectable marker (Km^R^/Nm^R^), and an *E. coli oriV* (p15A). Capture of this plasmid in Rif^R^ *E. coli* DH5αR by plating the mating spots on LB with Rif and Km proved challenging due to background caused by the pRK600 plasmid reverting from Cm to Km resistance [109]. To better select for desired *E. coli* transconjugants containing the B108/B109/B123 regions, the previous observation that *S. meliloti* thiamine synthesis genes could complement *E. coli* thiamine auxotrophs was utilized [109]. As the DH5α strain of *E. coli* is a thiamine auxotroph and the *S. meliloti* thiamine synthesis genes are in the B123 region of pSymB, successful capture of the large, excised plasmid from RmP4242 in *E. coli* DH5αR resulted in thiamine prototrophy. A colony which grew on M9 glucose without thiamine was purified and confirmed to contain the B108/B109/B123 regions via colony PCR.

Once the B108/B109/B123 region was captured as a plasmid (pTH3247), it was re-introduced into a suitable *S. meliloti* background strain, RmP3952, that had pSymB removed along with the ETR region from *S. fredii* integrated into the chromosome (with *S. meliloti bacA* under its own promoter) [45]. For this integration, we employed *attP*/*attB* sites, which are recognized and recombined by the ΦC31 integrase as previously described [40]. As the B108/B109/B123-containing plasmid, pTH3247, possessed the *attP* inherited from pFL916 (see [136]), only the ΦC31 integrase gene and an *attB* site needed to be introduced into the chromosome of RmP3952. The ΦC31 integrase gene was transferred onto the chromosome of *S. meliloti* RmP110 along with an adjacent Nm^R^ cassette flanked by two *FRT* sites (strain RmP1684). This was subsequently transduced into *S. meliloti* RmP3952 and the Nm^R^ was removed via Flp expression from pTH2505 that was subsequently lost, yielded RmP4239. The next step involved the transfer of the *attB* site from the Gm^R^, *sacB* integrative plasmid pTH2287 as done previously [40] into *S. meliloti* RmP4239. This ΔpSymB RmP3952 derivative with both the ΦC31 integrase gene and an *attB* on the chromosome was purified and designated RmP4245. The *attB* site was positioned between *S. meliloti* chromosomal genes *smc03108* and *smc03107* immediately after nt 3,234,049. A triparental mating to transfer the plasmid pTH3247 harbouring the isolated B108/B109/B123 *S. meliloti* regions and an *attP* site on a Km^R^/Nm^R^ plasmid from *E. coli* DH5αR to *S. meliloti* RmP4245 (which lacks pSymB) was conducted by plating the mating spots on LB_mc_ with cobalt, and 0.5 mM IPTG to induce the ΦC31 integrase gene under control of the hybrid tac-promoter (Ptac) (Fig. S1B3). Selection for transconjugants in which pTH3247 was integrated into the chromosome of *S. meliloti* RmP4245 at the *attB* site was performed with Sm and Nm (Fig. S1B4). Transconjugants appeared to grow more rapidly than the ΔpSymB RmP4245 recipient and the presence of the B108/B109/B123 region in the newly purified strain, termed SmB1.0 (RmP4256), was subsequently confirmed via Illumina sequencing. *S. meliloti* RmP4256 was routinely confirmed via its Nm^R^ and its inability to utilize galactitol (dulcitol) as a sole carbon source [139].

### Construction of pSymBΔB302, its deletion derivatives, and re-introduction to SmB1.0

The previous section detailed the creation of pSymBΔB301 and the subsequent excision of the adjacent B108-B109-B123 regions as plasmid pTH3247 from the *S. meliloti* strain RmP4242. The remaining *S. meliloti* strain and its remaining pSymB, once cured of the *flp* expression plasmid pTH2505, was termed RmP4369 and contained a deletion of pSymB nt 1,131,169–1,652,557 (ΔB302).

The pSymBΔB302 of RmP4369 contained a deletion scar that included an *oriT* and a Gm^R^ gene. This enabled us to transfer pSymBΔB302 to SmB1.0 using *E. coli* MT616 as a helper and selecting for transconjugants on M9 without thiamine and with 10 mM succinate (both of which select for SmB1.0 recipients as RmP4369 is missing both the *thi* and *dct* genes) as well as Gm to select for pSymBΔB302 itself. The resultant strain, RmP4370, added back the remaining pSymB genes to SmB1.0 except for genes within the B301 region that were not complemented by the *S. fredii* ETR genes on the chromosome.

Deletions within pSymBΔB302 were made by transducing existing Nm^R^/Gm^R^ marked deletions into RmP4369 (selecting for Nm^R^) and then transferring the resultant pSymB to SmB1.0 as before. This was performed to create strains RmP4550-RmP4558 (see Additional file 7: Table S3). In all instances, the individual deletions were made by employing the same workflow of transferring two integrative plasmids containing *FRT* sites into pSymB flanking the region to be deleted and recombining the *FRT* sites with Flp recombinase [113].

### Construction of *S. meliloti* strain SmB2.0

Construction of a minimized, replicating pSymB began with taking wild-type RmP110 and integrating *FRT* site-containing plasmids pFL3953 (Gm^R^) [136] and pTH1994 (Nm^R^) [113] into pSymB via single homologous recombination to make strain RmP877. The region of pSymB DNA between the two *FRT* sites (nt 122,109–466498) was then deleted via the expression of Flp recombinase from pTH2505, which was subsequently lost, yielding RmP2718 (ΔB142). This strain was subsequently transduced with RmP2719 to add the essential *engA* and *tRNA*^ARG^ genes from pSymB to the chromosome, which was selected for with Sp [43], to give RmP2720 [29].

The strain RmP2720 was next transduced with RmP2745, a strain deleted in pSymB nt 635,941–869,641 (ΔB180). RmP2745 itself was constructed by transducing the Gm^R^/Nm^R^ marked deletion of pSymB nt 635,941–678,811 (ΔB143) from strain RmP1108 into the strain RmP874 that contained the unmarked deletion of pSymB nt 743,315–869,641 (ΔB139) and subsequently deleting the additional region between the two *FRT* sites via Flp recombinase [29]. It should be noted here that a single *FRT* site remains after a deletion, which allows for the transduction of one marked deletion into a strain with an unmarked deletion to flank a larger region to be deleted with two *FRT* sites. The resultant strain, RmP3557 (RmP2720 Φ RmP2745), contained two *FRT* sites that were then once more recombined via Flp recombinase from pTH2505 to delete pSymB nt 122,109–869641 and give rise to the unmarked deletion strain RmP3558 (ΔB201) [29]. The integrative plasmid pTH1938 (Nm^R^) was then subsequently integrated into RmP3558 via single homologous recombination of pSymB nt 1,129,758–1,131,168 to introduce a second *FRT* site. Another round of Flp-mediated recombination of the two *FRT* sites deleted the additional pSymB DNA that brought the total deleted region to pSymB nt 122,109–1129757 (ΔB310), forming strain RmP3560 (SmB2.0). Its pSymB content was termed minSymB2.0.

## Supplementary Information

Additional file 1: Table S1. *S. meliloti* orthologs of *S. fredii* ETR region genes as determined by RBH analysis.

Additional file 2: Fig. S1. Schematic for capture of pSymB regions B108, B109, and B123 and creation of SmB1.0 via their integration into a ΔpSymB background strain. A. (1) Conjugal transfer and subsequent integration of two integrative plasmids harbouring mutant *loxP* sites to the right flank of B109 (purple) and the left flank of B123 (blue) into a strain of *S. meliloti* in which the *S. fredii* ETR region has been integrated into the chromosome (orange). (2) Transduction of a promoterless *cre* integrative plasmid into pSymB downstream of the taurine inducible promoter P_*tauA*_. (3) Resultant strain now contains *loxP* sites flanking the B301 region (yellow) to be deleted, that are recombined by Cre which is expressed by the addition of 10 mM taurine. (4) Conjugal transfer and subsequent integration of two integrative plasmids harbouring *FRT* sites to the left flank of B108 (red) and the right flank of B123 (blue) of the now adjacent B108/B109/B123 symbiotic regions. B. (1) Conjugal transfer of the PCA-inducible Flp expression plasmid pTH2505 into the *S. meliloti* recipient with *FRT* sites flanking B108/B109/B123. (2) Triparental mating and concurrent expression of Flp in the now *S. meliloti* donor with an *E. coli* recipient DH5αR. (3) Captured B108/B109/B123 regions in *E. coli* are re-introduced into a ΔpSymB recipient via conjugation and subsequently integrated into the chromosome at an *attB* landing pad via chromosomally expressed ΦC31 integrase. (4) Resultant minSymB1.0 strain with the *S. fredii* ETR region and B108/B109/B123 regions integrated into the chromosome.

Additional file 3: Fig. S2. Effect of the addition of cobalt on the kinetics of nodules formation by *S. meliloti* SmB1.0 on alfalfa seedlings. (A) When Jensen’s agar omits the 2 µM CoCl_2_ supplementation, the proportion of nodulated plants is reduced for SmB1.0-inoculated plants (light red). The addition of cobalt restores this to a wild-type rate of nodulation (dark red). (B) Compared to Fig. 2C, the absence of cobalt in the Jensen’s agar slants causes nodules to emerge later, and in smaller overall numbers for plants inoculated with SmB1.0. Data is the average of 20 plants for each condition. Shaded regions indicate standard deviation. Cobalt had no effect on nodulation kinetics of plants inoculated with the wild type.

Additional file 4: Fig. S3. Visual summary of workflow conducted to vegetatively propagate alfalfa cuttings. Initial plants were noted for their height and then cut-back to above the bottom node. Media in the bottom jar was replaced with fresh Jensen’s containing 10 mM nitrate and plants were allowed to re-grow under propagation domes. Cut-back was repeated three times. Cuttings were then taking that ranged from 5-10 cm in length that contained at least two nodes, leaves were removed, and the lowest petiole was dipped in 0.1% IBA before being buried in new sand/vermiculite containing Jensen’s media (no nitrogen). After initial rooting for two weeks under propagation domes, cuttings were inoculated with *S. meliloti* and allowed to grow for another five weeks, whereupon the shoot heights were observed.

Additional file 5: Table S2. Additional germplasm of *M. sativa* used to assess the SNF capacity of SmB1.0.

Additional file 6: Fig. S4. Symbiotic phenotype of key strains on the alternate *S. meliloti* host *Melilotus officinalis* (yellow sweet clover) and the consistent nodule morphology of exclusively Fix^−^ SmB1.0 nodules. (A) The shoot dry weight of *M. officinalis* inoculated with SmB1.0 was consistently Fix^−^ and indistinguishable from un-inoculated controls (red). This appears to be due to the simultaneous removal of the B301 and B307 regions as seen when *M. officinalis* is inoculated with SmB1.0+ pSymBΔB302ΔB307. The addition of pSymBΔB302 to SmB1.0 was able to restore the symbiosis wild-type levels as was seen with alfalfa (light blue). SmB2.0 was Fix^+^ with *M. officinalis* at a level below that seen with alfalfa (orange). Mean shoot height differences were assessed using one-way ANOVA followed by a Tukey multiple comparison test. (B-D) Wild-type-inoculated nodules of *M. officinalis* 5 weeks post-inoculation displayed typical development with highly elongated bacteroids. (E-J) SmB1.0-inoculated nodules of *M. officinalis* 5 weeks post-inoculation exhibited consistent early arrest of nodule development (E-F, further magnified in G) with poorly elongated bacteria (H). Proximal part of the nodule was often devoid of infected cells and possessed numerous infection threads (I-J). Nodules were fixed and stained with the DNA-binding dye SYTO13. Images are representative of a minimum of five independent nodules examined. Scale bars: (B, E-G) 100 μm; (C-D, H-J) 25 μm. Arrowheads point to examples of bacteria in infection threads.

Additional file 7: Table S3. Strains and plasmids used in this study.

Additional file 8: Table S4. Primers used in this study.

Additional file 9: Fig. S5. Phenotypic assessment of alfalfa nodules for SmB1.0 grown in Leonard jars with and without exogenous cobalt. Plants were grown for 35 days in a sand/vermiculite medium with Jensen’s solution with and without 2 μM cobalt. (A) Number of nodules present on each plant (n = 18) for wild type, SmB1.0, and a strain lacking pSymB entirely (ΔpSymB) grown with and without additional cobalt. Only a slight increase in nodules for SmB1.0 with cobalt was observed. (B) Nodule fresh weight averaged per nodule per plant (n = 18) inoculated with either wild type or SmB1.0. No differences were observed between the treatments. (C) Shoot dry weight of alfalfa plants inoculated with strains missing the *cbtJKL* operon showed little difference with the addition of cobalt to the Jensen’s solution. Assessments were made using one-way ANOVA with a Šidák correction. *p < 0.05.

## Data Availability

No datasets were generated or analysed during the current study.

## Abbreviations

BNF: Biological nitrogen fixation
SNF: Symbiotic nitrogen fixation
EPS: Exopolysaccharide
ETR: *engA*-tRNA_ARG_-*rmlC* region
*Sf*: *Sinorhizobium fredii*
*Sm*: *Sinorhizobium meliloti*
SDW: Shoot dry weight
ARA: Acetylene reduction assay
PI: Propidium iodide
NCR: Nodule-specific cysteine-rich
LMW: Low molecular weight
HMW: High molecular weight
Sm: Streptomycin
Sp: Spectinomycin
Nm: Neomycin
Gm: Gentamicin
Tc: Tetracycline
Cm: Chloramphenicol
Rif: Rifampicin
Km: Kanamycin
IBA: Indole-3-butyric acid
PBS: Phosphate-buffered saline
RBH: Reciprocal best hits
CTAB: Cetyltrimethylammonium bromide
PCA: Protocatechuic acid
