# Supplementary figures and images for: A minimized symbiotic gene set from the 1.68 Mb pSymB chromid of Sinorhizobium meliloti reveals auxiliary symbiotic loci
Source: BMC Biol. 2025 Jul 9;23:204. doi: 10.1186/s12915-025-02298-5 (PMC12239276; doi:10.1186/s12915-025-02298-5)

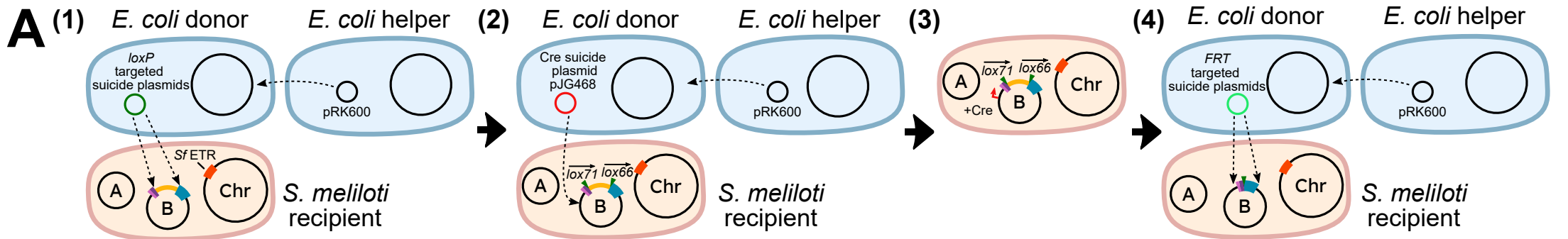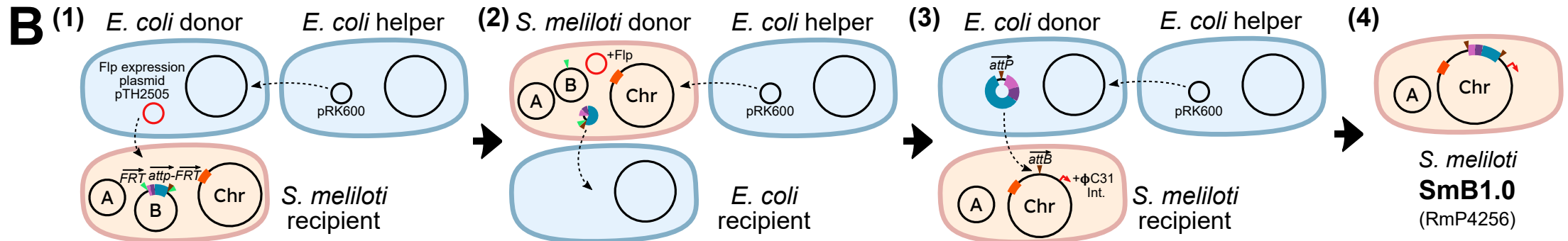

Supplement: Supplementary file 1 — Additional file 1: Table S1. S. meliloti orthologs of S. fredii ETR region genes as determined by RBH analysis. [file 12915_2025_2298_MOESM1_ESM.pdf]

**A**

% Plants with nodules

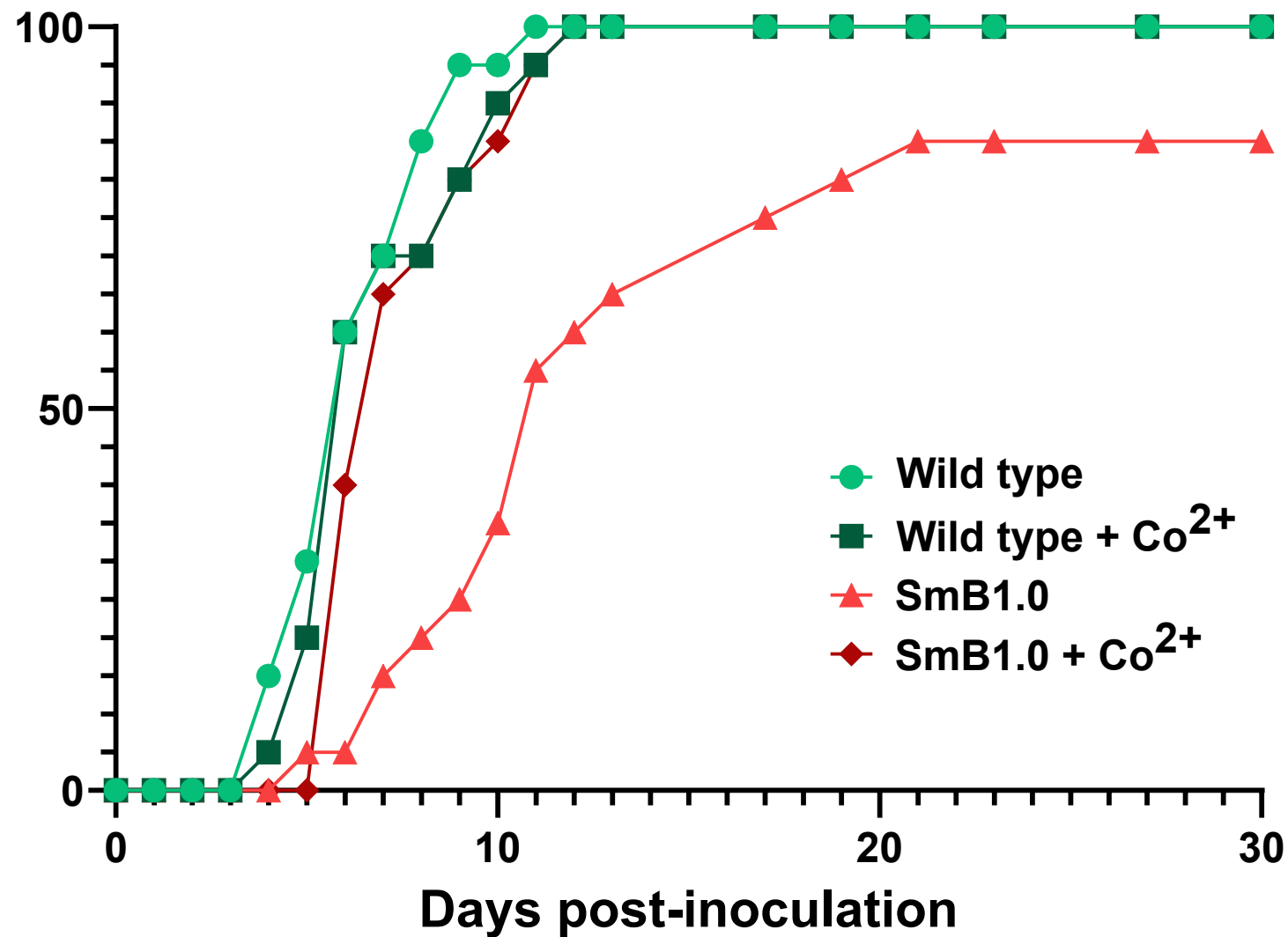**B**

Nodules per plant

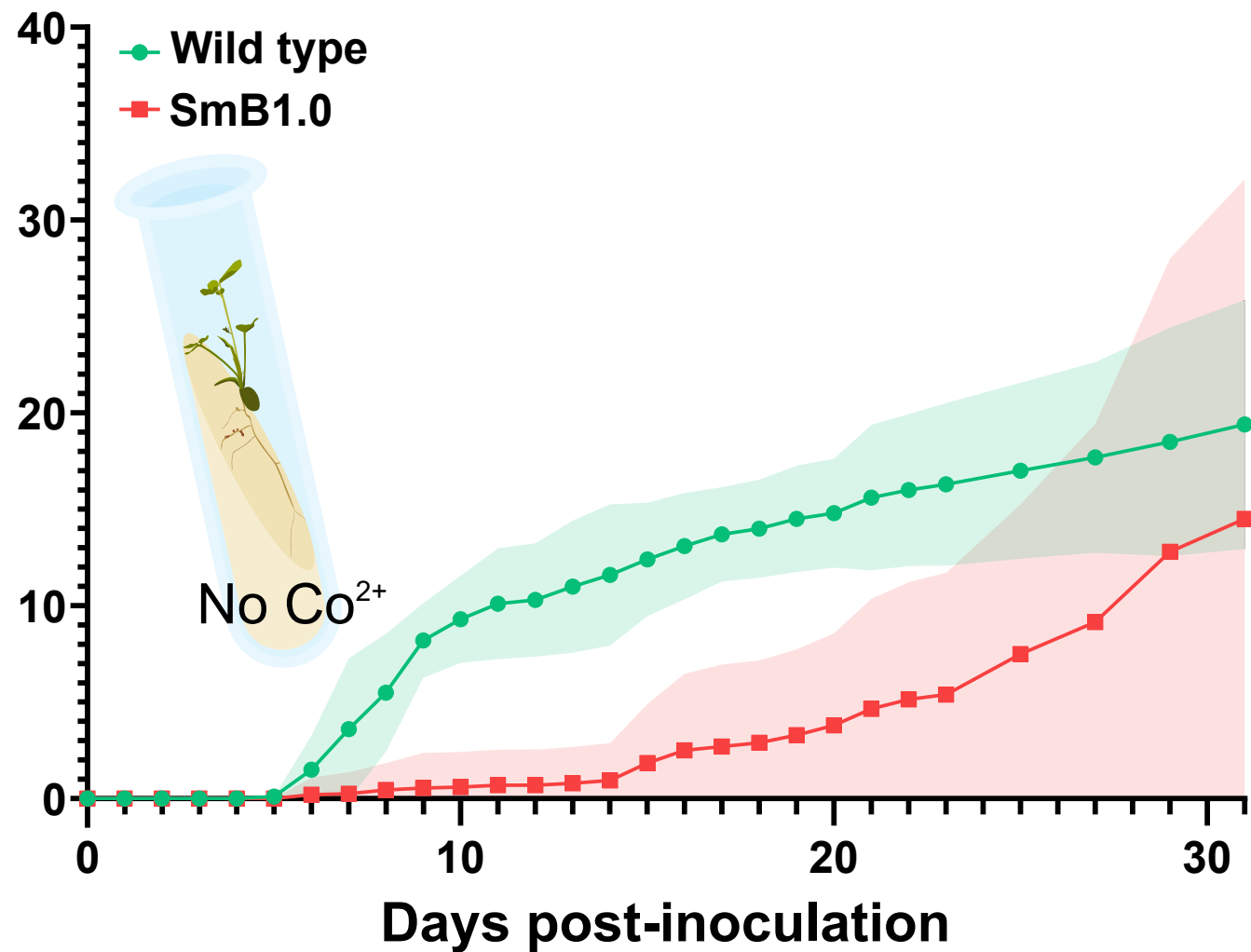

Supplement: Supplementary file 2 — Additional file 2: Fig. S1. Schematic for capture of pSymB regions B108, B109, and B123 and creation of SmB1.0 via their integration into a ΔpSymB background strain. A. (1) Conjugal transfer and subsequent integration of two integrative plasmids harbouring mutant loxP sites to the right flank of B109 (purple) and the left flank of B123 (blue) into a strain of S. meliloti in which the S. fredii ETR region has been integrated into the chromosome (orange). (2) Transduction of a promoterless cre integrative plasmid into pSymB downstream of the taurine inducible promoter PtauA. (3) Resultant strain now contains loxP sites flanking the B301 region (yellow) to be deleted, that are recombined by Cre which is expressed by the addition of 10 mM taurine. (4) Conjugal transfer and subsequent integration of two integrative plasmids harbouring FRT sites to the left flank of B108 (red) and the right flank of B123 (blue) of the now adjacent B108/B109/B123 symbiotic regions. B. (1) Conjugal transfer of the PCA-inducible Flp expression plasmid pTH2505 into the S. meliloti recipient with FRT sites flanking B108/B109/B123. (2) Triparental mating and concurrent expression of Flp in the now S. meliloti donor with an E. coli recipient DH5αR. (3) Captured B108/B109/B123 regions in E. coli are re-introduced into a ΔpSymB recipient via conjugation and subsequently integrated into the chromosome at an attB landing pad via chromosomally expressed ΦC31 integrase. (4) Resultant minSymB1.0 strain with the S. fredii ETR region and B108/B109/B123 regions integrated into the chromosome. [file 12915_2025_2298_MOESM2_ESM.pdf]

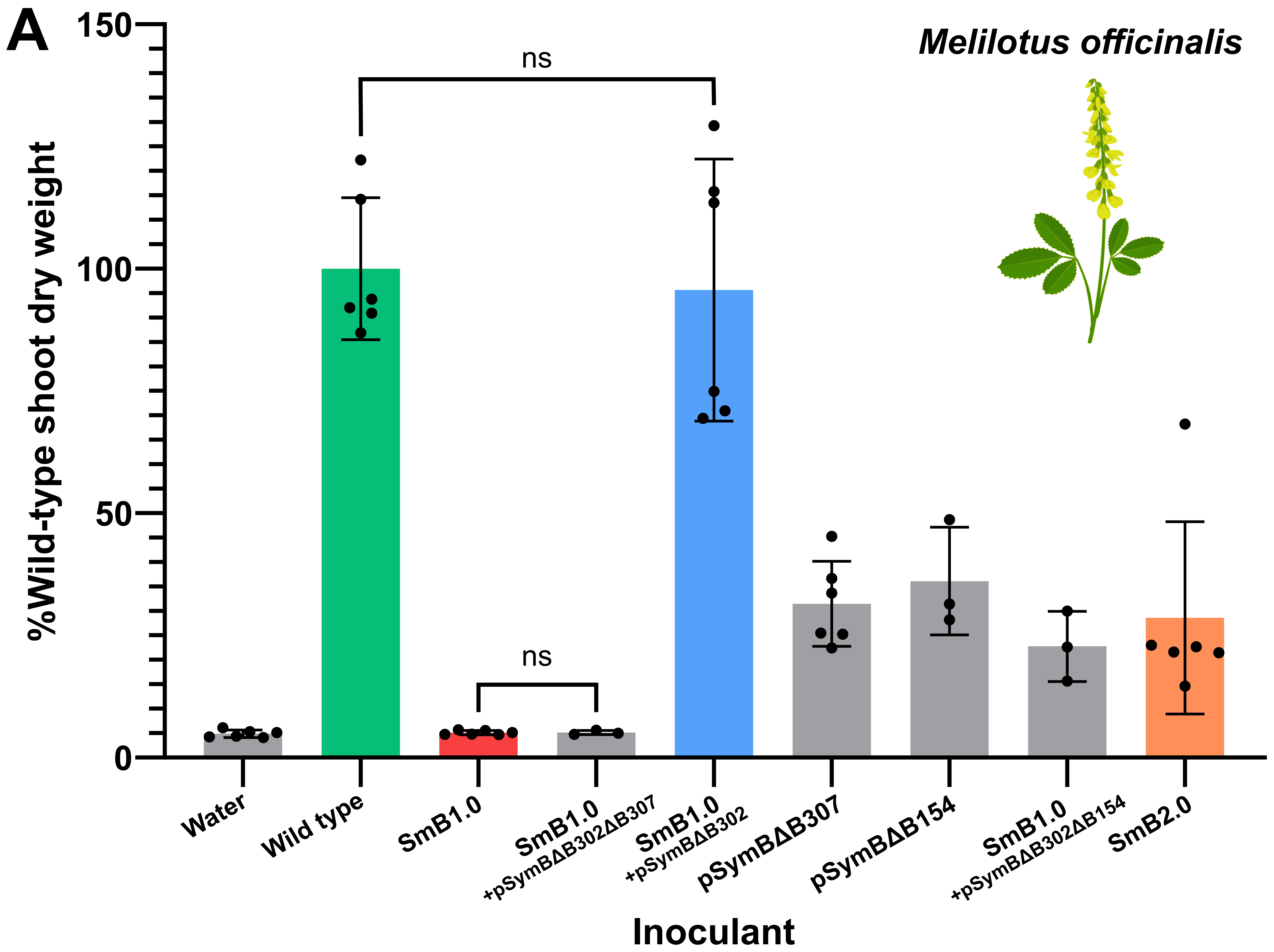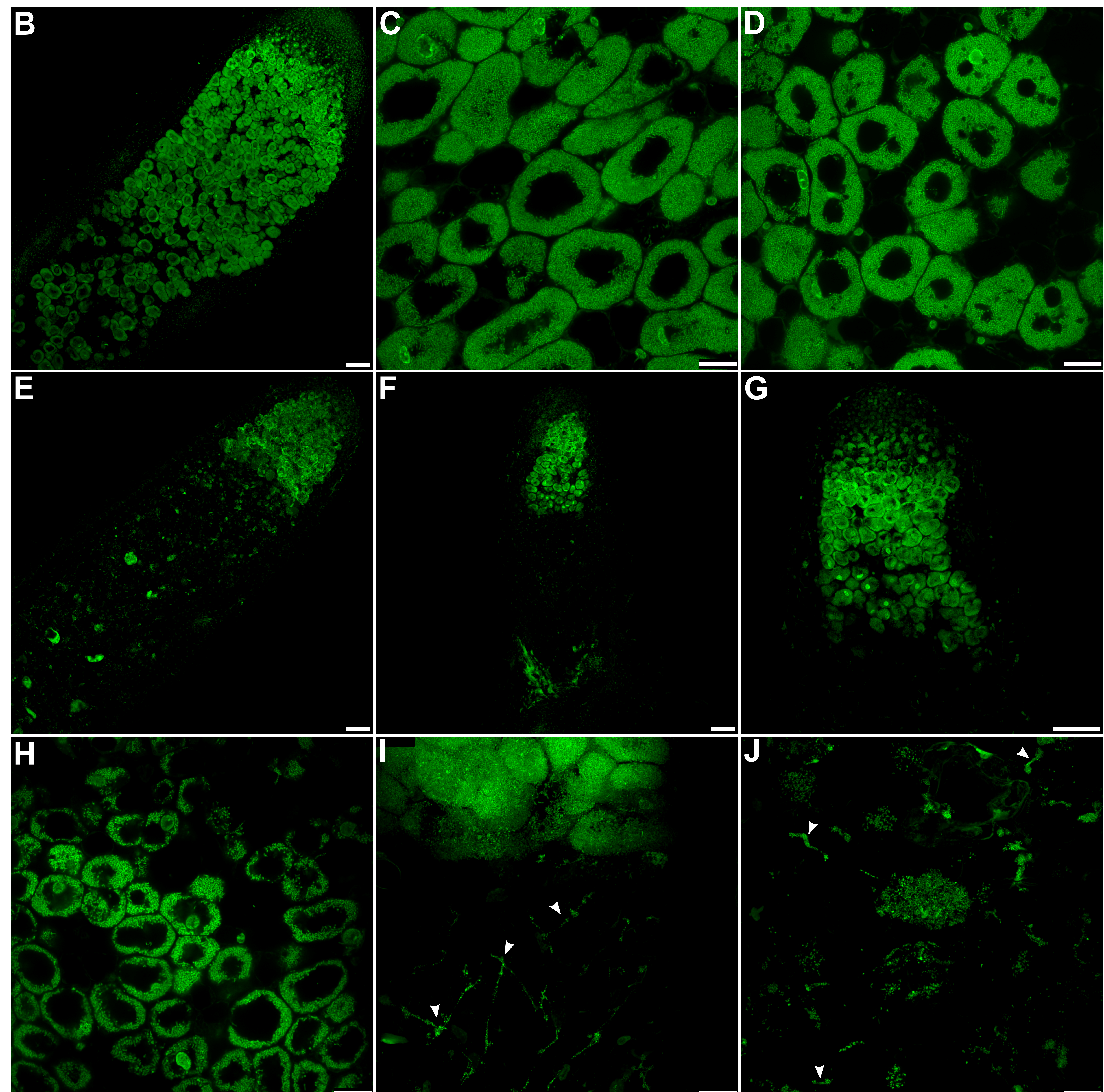

Supplement: Supplementary file 4 — Additional file 4: Fig. S3. Visual summary of workflow conducted to vegetatively propagate alfalfa cuttings. Initial plants were noted for their height and then cut-back to above the bottom node. Media in the bottom jar was replaced with fresh Jensen’s containing 10 mM nitrate and plants were allowed to re-grow under propagation domes. Cut-back was repeated three times. Cuttings were then taking that ranged from 5-10 cm in length that contained at least two nodes, leaves were removed, and the lowest petiole was dipped in 0.1% IBA before being buried in new sand/vermiculite containing Jensen’s media (no nitrogen). After initial rooting for two weeks under propagation domes, cuttings were inoculated with S. meliloti and allowed to grow for another five weeks, whereupon the shoot heights were observed. [file 12915_2025_2298_MOESM4_ESM.pdf]

**A**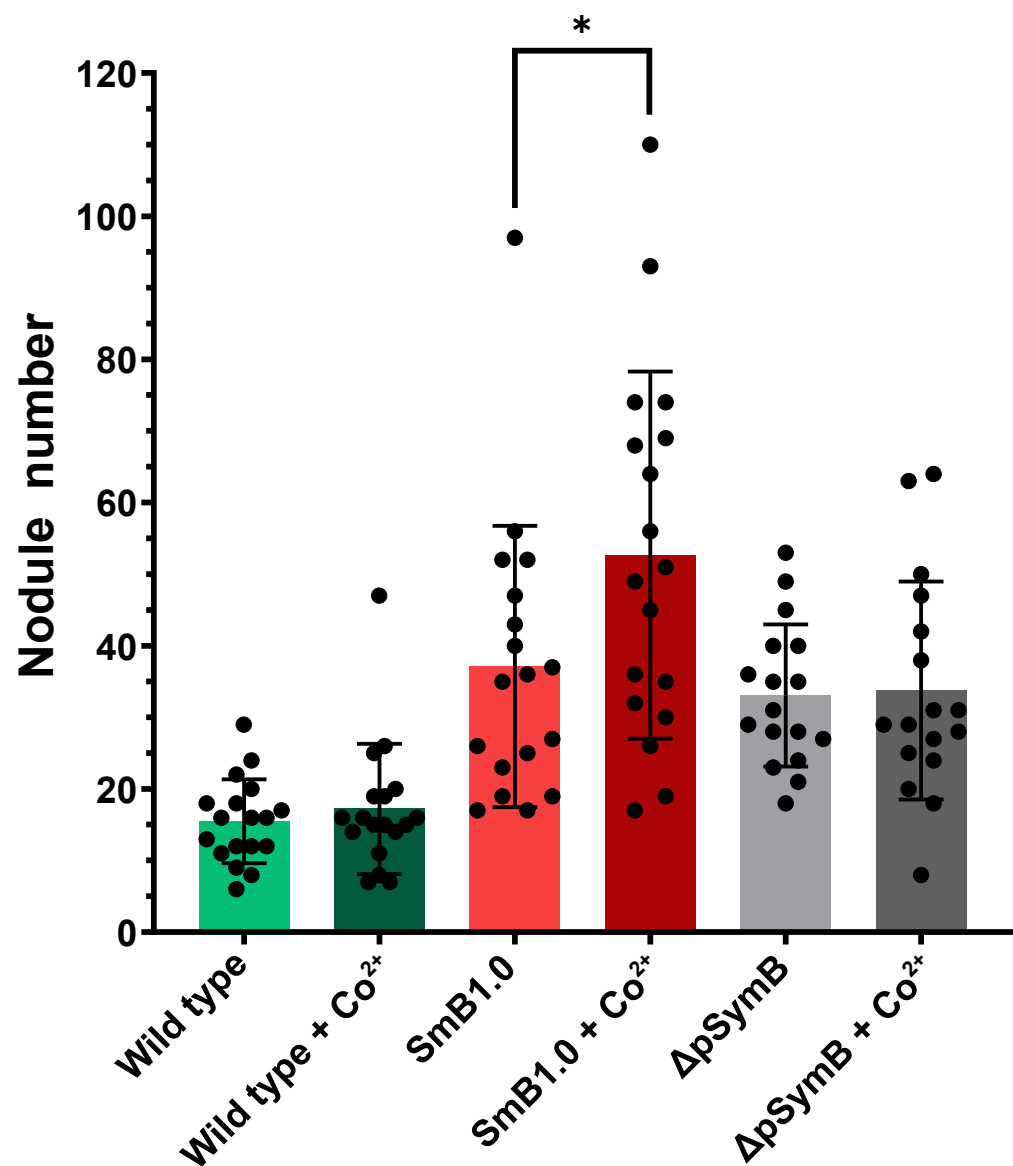**B**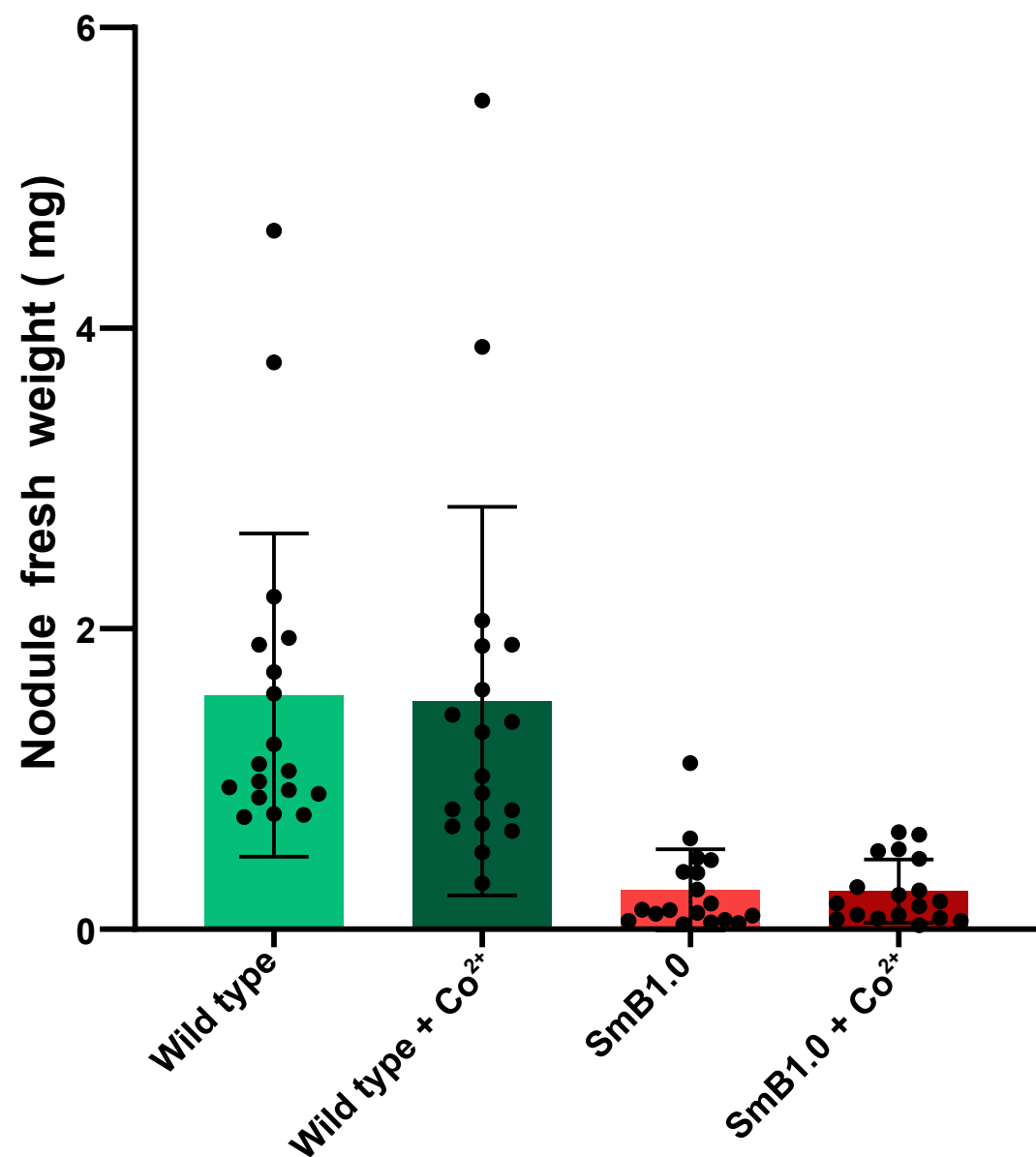**C**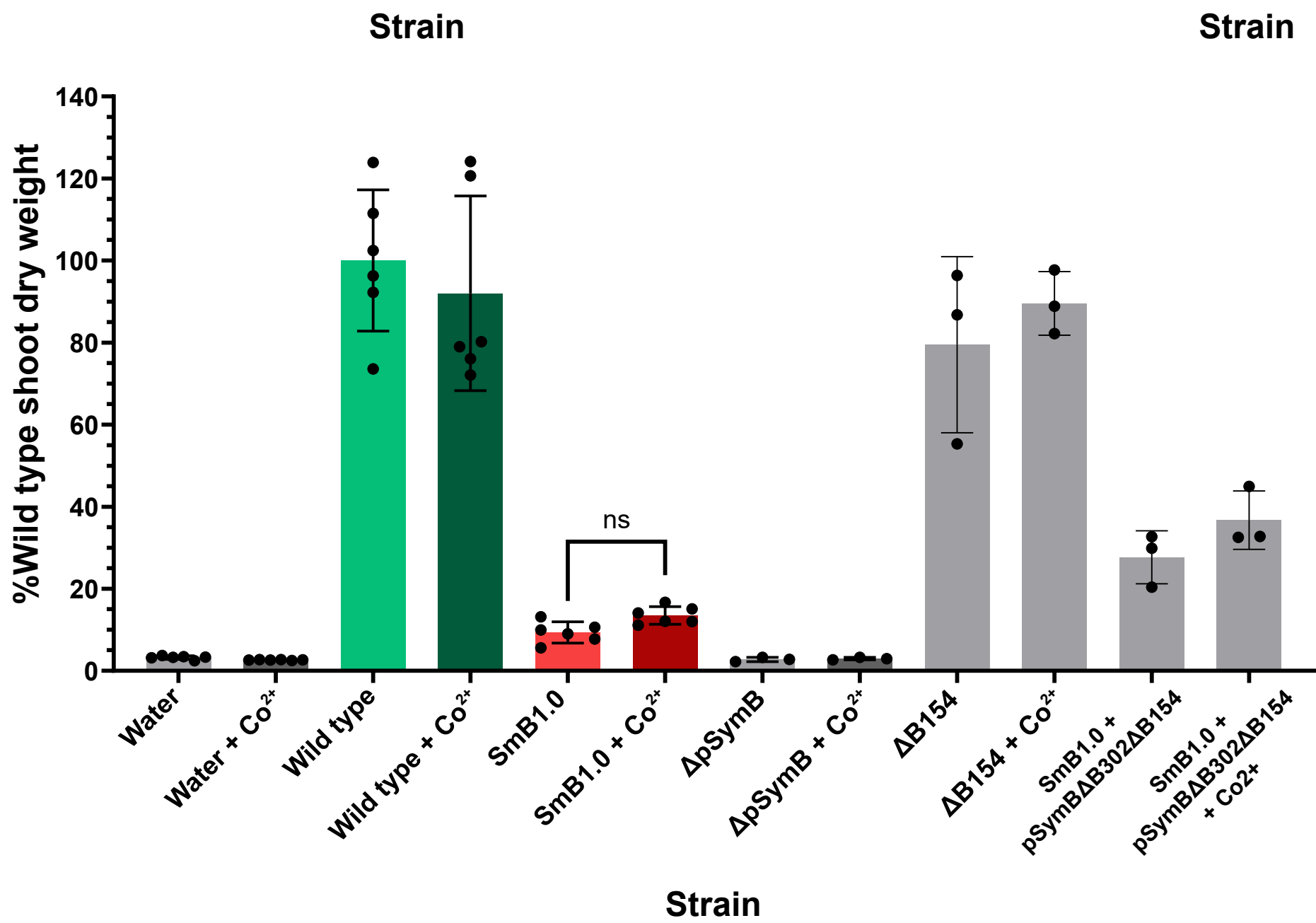

Supplement: Supplementary file 5 — Additional file 5: Table S2. Additional germplasm of M. sativa used to assess the SNF capacity of SmB1.0. [file 12915_2025_2298_MOESM5_ESM.pdf]
