# Supplementary material for: A minimized symbiotic gene set from the 1.68 Mb pSymB chromid of Sinorhizobium meliloti reveals auxiliary symbiotic loci
Source: BMC Biol. 2025 Jul 9;23:204. doi: 10.1186/s12915-025-02298-5 (PMC12239276; doi:10.1186/s12915-025-02298-5)
Supplement: Supplementary file 8 — Additional file 8: Table S4. Primers used in this study. [file 12915_2025_2298_MOESM8_ESM.docx]

**Table S3. Strains and plasmids used in this study.**

| Strain/Plasmid | Characteristics | Reference |
| --- | --- | --- |
| *Sinorhizobium meliloti* |  |  |
| Rm2011 | Wild type SU47 *str-3*; Sm^R^ | [137] |
| RmP110 | Rm1021 Wild type SU47 *str-21* with corrected wild type *pstC*; Sm^R^ | [50] |
| RmG488 | Rm7031 – Rm1021 *exoA31*::Tn5; Sm^r^, Nm^R^ | [117] |
| RmP796 | RmP110-derived strain with deletion of pSymB nt 71018-752547 (ΔB311); Sm^R^, Nm^R^, Gm^R^ | Finan lab collection |
| RmP874 | RmP110 Ω pFL203 via pSymB nt 741497- 743314, Ω pTH1939 via pSymB nt 869642- 870505 and subsequent deletion of pSymB nt 743315-869641 (ΔB139) via Flp-mediated recombination of FRT sites; Sm^R^, Tc^R^ | [111] |
| RmP877 | RmP110 Ω pFL3953 via pSymB nt 121311-122108, Ω pTH1994 via pSymB nt 466499-467160; Sm^R^, Gm^R^, Nm^R^ | Finan lab collection |
| RmP1059 | RmP110-derived strain with deletion of pSymB nt 62138-100635 (ΔB154); Sm^R^, Nm^R^, Gm^R^ | [29] |
| RmP1108 | RmP110 Ω pFL4178 via pSymB nt 678812- 679659, Ω pTH1995 via pSymB nt 635019-635940 and subsequent deletion of pSymB nt 635941-678811 (ΔB143) via Flp-mediated recombination of FRT sites; Sm^R^, Gm^R^, Nm^R^, Tc^R^ | [111] |
| RmP1109 | RmP110-derived strain with deletion of pSymB nt 635941-744319 (ΔB145); Sm^R^, Nm^R^, Gm^R^ | Finan lab collection |
| RmP1684 | RmP110 with *ΦC31* integrase on the chromosome; Sm^R^, Nm^R^ | Finan lab collection |
| RmP2707 | RmP110-derived strain with deletion of pSymB nt 1679724-49522 (ΔB161); Sm^R^, Nm^R^, Gm^R^ | This study |
| RmP2710 | RmP110-derived strain with deletion of pSymB nt 740723-869641 (ΔB305); Sm^R^, Nm^R^, Gm^R^ | This study |
| RmP2713 | RmP110-derived strain with deletion of pSymB nt 101397-345340 (ΔB306); Sm^R^, Nm^R^, Gm^R^ | This study |
| RmP2714 | RmP110-derived strain with deletion of pSymB nt 101397-220004 (ΔB312); Sm^R^, Nm^R^, Gm^R^ | This study |
| RmP2716 | RmP110-derived strain with deletion of pSymB nt 870506-1129757 (ΔB307); Sm^R^, Nm^R^, Gm^R^ | This study |
| RmP2717 | RmP110-derived strain with deletion of pSymB nt 451558-651862 (ΔB308); Sm^R^, Nm^R^, Gm^R^ | This study |
| RmP2718 | RmP877 with deletion of pSymB nt 122109-466498 (ΔB142) via lost pTH2505; Sm^R^ | Finan lab collection |
| RmP2719 | RmP110 with Sp^R^-marked chromosomal insertion of the *engA* and tRNA^ARG^ genes from pSymB; Sm^R^, Sp^R^ | [43] |
| RmP2720 | RmP2718 Φ RmP2719; Sm^R^, Sp^R^ | [29] |
| RmP2745 | RmP874 Φ RmP1108 with deletion of pSymB nt 635941-869641 (ΔB180) via Flp-mediated recombination of FRT sites; Sm^R^, Nm^R^, Tc^R^ | [111] |
| RmP3557 | RmP2720 Φ RmP2745; Sm^R^, Sp^R^, Nm^R^ | [29] |
| RmP3558 | RmP3557 with deletion of pSymB nt 122109-869641 (ΔB201) via Flp-mediated recombination of FRT sites from lost pTH2505; Sm^R^, Sp^R^ | [29] |
| RmP3559 | RmP3558 Ω pTH1938 via pSymB nt 1129758-1131168; Sm^R^, Sp^R^, Nm^R^ | This study |
| RmP3560  (SmB2.0) | RmP3559 with deletion of pSymB nt 122109-1129757 (ΔB310) via Flp-mediated recombination of FRT sites from lost pTH2505; Sm^R^, Sp^R^ | This study |
| RmP3952 | Rm2011 ΩETR(*Sf*) Δ*bacA*(*Sf*)::*bacA*(*Sm*) ΔpSymB – Rm2011 with pSymB removed and the *engA-*tRNA^arg^-*rmlC* region from *Sinorhizobium fredii* NGR234 with *S. meliloti bacA* gene and promoter on the chromosome; Sm^R^ | [45] |
| RmP3957 | Rm2011 (ΔpSymB) with the *engA-*tRNA^arg^-*rmlC* region from *Sinorhizobium fredii* NGR234 with *S. meliloti bacA* gene on the chromosome; Sm^R^ | [45] |
| RmP4224 | RmP3957 Ω pTH3242 via pSymB nt 1530068-1530558; Sm^R^, Nm^R^ | This study |
| RmP4226 | RmP110 Ω pJG468 via pSymB nt 1053369-1053819; Sm^R^, Tc^R^ | This study |
| RmP4227 | RmP4224 Ω pTH3244 via pSymB nt 1203758-1204420; *sacB*, Sm^R^, Nm^R^, Gm^R^ | This study |
| RmP4232 | RmP4227 ΦRmP4226; *sacB*, Sm^R^, Nm^R^, Gm^R^, Tc^R^ | This study |
| RmP4233 | RmP4227 after taurine induced Cre-mediated recombination of *lox66* and *lox71*. Deletion of pSymB nt 1204421-1530067 (ΔB301); Sm^R^, Tc^R^ | This study |
| RmP4234 | RmP4233 cured of pJG468 integrant. Deletion of pSymB nt 1204421-1530067 (ΔB301); Sm^R^ | This study |
| RmP4235 | RmP3952 Φ RmP1684; Sm^R^, Nm^R^ | This study |
| RmP4239 | RmP4235 after PCA induced Flp-mediated recombination of FRT sites flanking the Nm^R^ cassette and pTH2505 curing; Sm^R^ | This study |
| RmP4240 | Rmp4234 ΔB301 Ω pTH3243 via pSymB nt 1129758-1131168); Sm^R^, Nm^R^ | This study |
| Rmp4241 | RmP4240 Ω pFL916 via pSymB nt 1652558-1654191; Sm^R^, Nm^R^, Gm^R^ | This study |
| RmP4242 | RmP4241 (pTH2505); Sm^R^, Nm^R^, Gm^R^, Tc^R^ | This study |
| RmP4243 | RmP4239 Ω pTH2287 via chromosome nt 3233592-3234473; Sm^R^, Gm^R^) | This study |
| RmP4245 | RmP4243 (pTH2287 double recombinant), introduces attB via SmaI site on chromosome nt 3234050; Sm^R^ | This study |
| RmP4256  (SmB1.0) | RmP4245 Ω pTH3247 via IPTG-induced attP/attB recombination; Sm^R^, Nm^R^ | This study |
| RmP4369 | RmP4242 post-*flp* expression from lost pTH2505 plasmid. Deletion of pSymB nt 1131169-1652557 (ΔB302); Sm^R^, Gm^R^ | This study |
| RmP4370 | RmP4256 with pSymB from RmP4369 – minSymB1.0 with pSymBΔB302; Sm^R^, Nm^R^, Gm^R^ | This study |
| RmP4530 | RmP4369 Ω pTH1939 via pSymB nt 869642- 870505; Sm^R^, Nm^R^, Gm^R^ | This study |
| RmP4541 | RmP4369 Φ RmP796 (ΔB311); Sm^R^, Gm^R^, Nm^R^ | This study |
| RmP4542 | RmP4369 Φ RmP1059 (ΔB154); Sm^R^, Gm^R^, Nm^R^ | This study |
| RmP4543 | RmP4369 Φ RmP1109 (ΔB145); Sm^R^, Gm^R^, Nm^R^ | This study |
| RmP4544 | RmP4369 Φ RmP2707 (ΔB161); Sm^R^, Gm^R^, Nm^R^ | This study |
| RmP4545 | RmP4369 Φ RmP2710 (ΔB305); Sm^R^, Gm^R^, Nm^R^ | This study |
| RmP4546 | RmP4369 Φ RmP2713 (ΔB306); Sm^R^, Gm^R^, Nm^R^ | This study |
| RmP4547 | RmP4369 Φ RmP2714 (ΔB312); Sm^R^, Gm^R^, Nm^R^ | This study |
| RmP4548 | RmP4369 Φ RmP2717 (ΔB308); Sm^R^, Gm^R^, Nm^R^ | This study |
| RmP4549 | RmP4530 post-*flp* expression from lost pTH2505 plasmid. Deletion of pSymB nt 870506-1129757 (ΔB307); Sm^R^, Gm^R^, Nm^R^ | This study |
| RmP4550 | RmP4256 with pSymB from RmP4541 – minSymB1.0 with pSymBΔB302ΔB311; Sm^R^, Nm^R^, Gm^R^ | This study |
| RmP4551 | RmP4256 with pSymB from RmP4542 – minSymB1.0 with pSymBΔB302ΔB154; Sm^R^, Nm^R^, Gm^R^ | This study |
| RmP4552 | RmP4256 with pSymB from RmP4543 – minSymB1.0 with pSymBΔB302ΔB145; Sm^R^, Nm^R^, Gm^R^ | This study |
| RmP4553 | RmP4256 with pSymB from RmP4544 – minSymB1.0 with pSymBΔB302ΔB161; Sm^R^, Nm^R^, Gm^R^ | This study |
| RmP4554 | RmP4256 with pSymB from RmP4545 – minSymB1.0 with pSymBΔB302ΔB305; Sm^R^, Nm^R^, Gm^R^ | This study |
| RmP4555 | RmP4256 with pSymB from RmP4546 – minSymB1.0 with pSymBΔB302ΔB306; Sm^R^, Nm^R^, Gm^R^ | This study |
| RmP4556 | RmP4256 with pSymB from RmP4547 – minSymB1.0 with pSymBΔB302ΔB312; Sm^R^, Nm^R^, Gm^R^ | This study |
| RmP4557 | RmP4256 with pSymB from RmP4548 – minSymB1.0 with pSymBΔB302ΔB308; Sm^R^, Nm^R^, Gm^R^ | This study |
| RmP4558 | RmP4256 with pSymB from RmP4549 – minSymB1.0 with pSymBΔB302ΔB307; Sm^R^, Nm^R^, Gm^R^ | This study |
| *Escherichia coli* |  |  |
| DH5α | F^-^, *endA1*, *hsdR17*(r_k_^-^, m_k_^+^), *supE44*, *thi-1*, *λ^-^*, *recA1*, *gyrA96*, *relA1*, Φ80d*lacZ*ΔM15 | [138] |
| DH5αR | Rifampicin-resistant derivative of DH5α; Rif^R^ | [135] |
| MT607 | *pro-82, thi-1*, *hsdR17*, *supE44*, *recA56* | [109] |
| MT616 | MT607 (pRK600) Plasmid contains RK2 transfer genes; Cm^R^ | [109] |
| Plasmids |  |  |
| pFL203 | pTH1522 with pSymB nt 741497- 743314 via XhoI; Gm^R^ | [133] |
| pFL916 | pTH1522 with pSymB nt 1652558-1654191 via XhoI; Gm^R^ | [133] |
| pFL3468 | pTH1522 with pSymB nt 100636-101396 via XhoI; Gm^R^ | [133] |
| pFL3559 | pTH1522 with pSymB nt 852869- 853845 via XhoI; Gm^R^ | [133] |
| pFL3953 | pTH1522 with pSymB nt 121311-122108 via XhoI; Gm^R^ | [133] |
| pFL4178 | pTH1522 with pSymB nt 678812- 679659 via XhoI; Gm^R^ | [133] |
| pJG468 | Promoterless *cre* integration vector for P*_tauA_* on pSymB, pMB1 *oriV*, RK2 *oriT*; Tc^R^ | [131] |
| pJQ200SK | *sacB* suicide vector with partial *lacZα*; Gm^R^ | [139] |
| pLAFR1 library | *S. meliloti* DNA cloned into pLAFR1 cosmid vector; Tc^R^ | [74] |
| pTH1937 | pACYC177 derived FRT targeting vector, p15A *oriV*, RK2 *oriT*; Km^r^/Nm^R^ | [111] |
| pTH1938 | pTH1937 with pSymB nt 1129758-1131168 via SpeI/EcoRI; Km^r^/Nm^R^ | [111] |
| pTH1939 | pTH1937 with pSymB nt 869642- 870505 via SpeI/EcoRI; Km^r^/Nm^R^ | [111] |
| pTH1522 | Reporter vector containing an FRT site, pMB1 *oriV*/*oriT*; Gm^R^ | [133] |
| pTH1945 | pTH1522 with *nptII* replacement of *aacC4*; Km^R^/Nm^R^ | [133] |
| pTH1994 | pTH1937 with pSymB nt 466499-467160 via SpeI/EcoRI; Km^r^/Nm^R^ | [111] |
| pTH1995 | pTH1937 with pSymB nt 635019-635940 via SpeI/EcoRI; Km^r^/Nm^R^ | [111] |
| pTH2505 | Flp cassette from pTH1944 (*flp* under PCA inducible promoter) in the unstable vector pRK7813; Tc^R^ | [134] |
| pTH2287 | pJQ200uc1 with *S. meliloti* chromosome nt 3233592-3234473 containing *attB* @ SmaI via NotI; *sacB*, Gm^R^ | [40] |
| pTH3242 | pTH1945 with HR_2N (*lox66*) via KpnI in forward orientation; Km^R^/Nm^R^ | This study |
| pTH3243 | pTH1937 with pSymB nt 1129758-1131168 via KpnI/HindIII; Km^R^/Nm^R^ | This study |
| pTH3244 | pJQ200SK with HR_1N (*lox71*) via BamHI in forward orientation; *sacB*, Gm^R^ | This study |
| pTH3247 | Plasmid formed after Flp-mediated recombination between the FRT sites in RmP4242 containing B108, B109, B123 regions from *S. meliloti* 2011; Km^R^/Nm^R^ | This study |
