# Supplementary material for: A minimized symbiotic gene set from the 1.68 Mb pSymB chromid of Sinorhizobium meliloti reveals auxiliary symbiotic loci
Source: BMC Biol. 2025 Jul 9;23:204. doi: 10.1186/s12915-025-02298-5 (PMC12239276; doi:10.1186/s12915-025-02298-5)
Supplement: Supplementary file 9 — Additional file 9: Fig. S5. Phenotypic assessment of alfalfa nodules for SmB1.0 grown in Leonard jars with and without exogenous cobalt. Plants were grown for 35 days in a sand/vermiculite medium with Jensen’s solution with and without 2 μM cobalt. (A) Number of nodules present on each plant (n = 18) for wild type, SmB1.0, and a strain lacking pSymB entirely (ΔpSymB) grown with and without additional cobalt. Only a slight increase in nodules for SmB1.0 with cobalt was observed. (B) Nodule fresh weight averaged per nodule per plant (n = 18) inoculated with either wild type or SmB1.0. No differences were observed between the treatments. (C) Shoot dry weight of alfalfa plants inoculated with strains missing the cbtJKL operon showed little difference with the addition of cobalt to the Jensen’s solution. Assessments were made using one-way ANOVA with a Šidák correction. *p < 0.05. [file 12915_2025_2298_MOESM9_ESM.docx]

**Table S4. Primers used in this study.**

| Primer Name | Sequence (5’ to 3’) | Reference |
| --- | --- | --- |
| HR_1_Fwd  HR_1_Rev | GGATCCCGGATGATGTCGAGCACGG  GGATCCATAACTTCGTATAATGTATGCTATACGAACGGTACTGCTTCTTCGTCAATTCCG | This study |
| HR_1_FN  HR_1_RNfix | TCAGAAGGATCCCGGATGATGTCGAGC  GGCAGCGGATCCATAACTTCGTATAGCATACATTATACGAACGGTACTGCTTCTTCG | This study |
| HR_2_Fwd  HR_2_Rev | GGTACCATAACTTCGTATAATGTATGCTATACGAACGGTATGGCAGGACACCTATCTCGC  GGTACCGCACGGTCAGCCATAATTTGCG | This study |
| HR_2_FN  HR_2_RN | GCCAGCGGTACCATAACTTCGTATAATGTATGC  TTAGTAGGTACCGCACGGTCAGCC | This study |
| 1938F  1938R | ATTCATGGTACCCGATCTGCGGCGCGGTTGC  TACGTAAAGCTTGATCGTGGTGGCGGCAACC | This study |
| 4440_DF  4440_DR | GACGTGTCCTTGAGGATCGC  CGCACCAGCATCATCTTCGC | [111] |
| 4068_UF  4068_UR | CTCGTCACAGGTATCCAGCC  CAACGTCTGGATCTGCCTCG | [111] |
| 3347_UF  3347_UR | AGGTCGAGCGAGACATTGCC  GTCAGCATGTCCTCGTAGCG | [111] |
| ML-07-245  ML-07-246 | GTGGCGTGAGATCAGACGC  CGCTTGTCGTCTACTGACGC | Fink & Finan, unpublished |
| ML-07-1019  ML-07-1020 | GGCTTTCGGATGCTGGAGCC  GTCATGGACTTTCAGGACGG | [40] |
| 2611_UF  2611_UR | ACGACAACCTCTCCAACGGC  TCCCAGCCTAGAACCTGACG | [111] |
| 916_DF  916_DR | TTGACCTCTTCGAGCCTCGG  GAAACTGATGGCAGCGACGG | [111] |
