# Supplementary material for: A minimized symbiotic gene set from the 1.68 Mb pSymB chromid of Sinorhizobium meliloti reveals auxiliary symbiotic loci
Source: BMC Biol. 2025 Jul 9;23:204. doi: 10.1186/s12915-025-02298-5 (PMC12239276; doi:10.1186/s12915-025-02298-5)
Supplement: Supplementary file 3 — Additional file 3: Fig. S2. Effect of the addition of cobalt on the kinetics of nodules formation by S. meliloti SmB1.0 on alfalfa seedlings. (A) When Jensen’s agar omits the 2 µM CoCl2 supplementation, the proportion of nodulated plants is reduced for SmB1.0-inoculated plants (light red). The addition of cobalt restores this to a wild-type rate of nodulation (dark red). (B) Compared to Fig. 2C, the absence of cobalt in the Jensen’s agar slants causes nodules to emerge later, and in smaller overall numbers for plants inoculated with SmB1.0. Data is the average of 20 plants for each condition. Shaded regions indicate standard deviation. Cobalt had no effect on nodulation kinetics of plants inoculated with the wild type. [file 12915_2025_2298_MOESM3_ESM.pdf]

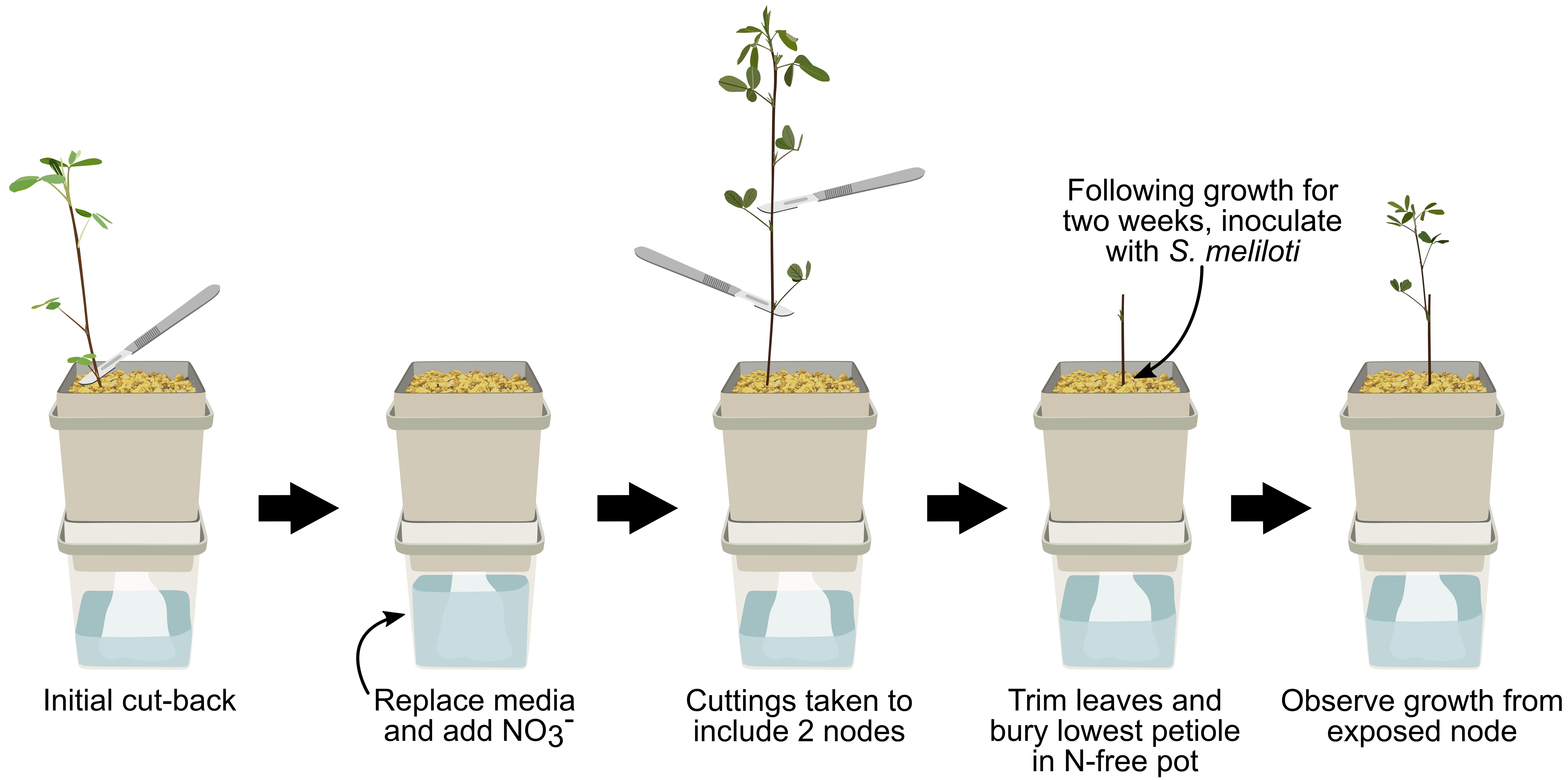
Following growth for
two weeks, inoculate
with *S. meliloti*
Initial cut-back
Replace media
and add NO_3^-
Cuttings taken to
include 2 nodes
Trim leaves and
bury lowest petiole
in N-free pot
Observe growth from
exposed node
